# NADPH Selective Depletion Nanomedicine‐Mediated Radio‐Immunometabolism Regulation for Strengthening Anti‐PDL1 Therapy against TNBC

**DOI:** 10.1002/advs.202203788

**Published:** 2022-11-20

**Authors:** Ying Wang, Di Gao, Lin Jin, Xuechun Ren, Yanan Ouyang, Ying Zhou, Xinyu He, Liangliang Jia, Zhongmin Tian, Dingcai Wu, Zhe Yang

**Affiliations:** ^1^ The Key Laboratory of Biomedical Information Engineering of Ministry of Education School of Life Science and Technology Xi'an Jiaotong University Xi'an 710049 China; ^2^ International Joint Research Laboratory for Biomedical Nanomaterials of Henan Zhoukou Normal University Zhoukou 466001 P. R. China; ^3^ PCFM Lab School of Chemistry Sun Yat‐sen University Guangzhou 510006 P. R. China; ^4^ Center of Accurate Diagnosis Treatment and Transformation of Bone and Joint Diseases The Eighth Affiliated Hospital Sun Yat‐sen University Shenzhen 518000 P. R. China

**Keywords:** drug‐like copolymer, immunometabolism regulation, immunosuppression of tumor microenvironment, low‐dose radiotherapy, selective depletion of NADPH

## Abstract

Anti‐PD(L)1 immunotherapy recently arises as an effective treatment against triple‐negative breast cancer (TNBC) but is only applicable to a small portion of TNBC patients due to the low PD‐L1 expression and the immunosuppressive tumor microenvironment (TME). To address these challenges, a multifunctional “drug‐like” copolymer that possesses the auto‐changeable upper critical solution temperature and the capacity of scavenging reduced nicotinamide adenine dinucleotide phosphate (NADPH) inside tumor cells is synthesized and employed to develop a hypoxia‐targeted and BMS202 (small molecule antagonist of PD‐1/PD‐L1 interactions)‐loaded nanomedicine (BMS202@HZP NPs), combining the anti‐PD‐L1 therapy and the low‐dose radiotherapy (LDRT) against TNBC. In addition to the controlled release of BMS202 in the hypoxic TNBC, BMS202@HZP NPs benefit the LDRT by upregulating the pentose phosphate pathway (PPP, the primary cellular source for NADPH) of TME whereas scavenging the NADPH inside tumor cells. As a result, the BMS202@HZP NPs‐mediated LDRT upregulate the PD‐L1 expression of tumor to promote anti‐PD‐L1 therapy response while reprogramming the immunometabolism of TME to alleviate its immunosuppression. This innovative nanomedicine‐mediated radio‐immunometabolism regulation provides a promising strategy to reinforce the anti‐PD‐L1 therapy against TNBC.

## Graphical abstract

A multifunctional “drug‐like” copolymer with the auto‐changeable upper critical solution temperature and selective depletion of reduced nicotinamide adenine dinucleotide phosphate (NADPH) inside tumor cells is synthesized and employed to develop a multifunctional nanomedicine (BMS202@HZP NPs), which sensitizes the low‐dose radiotherapy and achieves the radio‐immunometabolism regulation against triple‐negative breast cancer (TNBC), providing a promising strategy to reinforce anti‐PD‐L1 against TNBC.

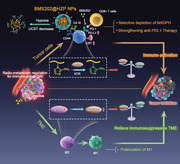

## 1 Introduction

According to the International Agency for Research on Cancer and the American Cancer Society, breast cancer makes the most common cancer diagnosed in women.^[^ ^1^ ^]^ Among different types of breast cancer, triple‐negative breast cancer (TNBC) possesses more aggressive invasiveness, higher metastasis rate, and more frequent recurrence, which leads to a consequence that the 5‐year relative survival rate for TNBC patients at late stage is only 14%. Additionally, the immune checkpoint blockade (ICB) anti‐PD‐(L)1 treatment only showed moderate efficacy in TNBC even in combination with chemotherapeutic drugs in the latest clinical trial,^[^ ^2^ ^]^ and its therapeutic efficacy is highly determined by the expression of PD‐L1 in TNBC.^[^ ^3^ ^]^ Given the fact that only 20% of the tumors overexpressed PD‐L1 due to the individual difference among TNBC patients,^[^ ^4^ ^]^ the treatment effectiveness of anti‐PD‐L1 therapy in clinical practice has been hindered. Moreover, the immune evasion caused by the tumor microenvironment (TME) also causes a reduction in the therapeutic effect of ICB.^[^ ^5^ ^]^ Therefore, a strategy that can promote the anti‐PD‐L1 therapy response while simultaneously alleviating the immunosuppression of TME would be valuable for improving the therapeutic efficacy of PD‐(L)1 blockade against TNBC.

**Figure 1:**
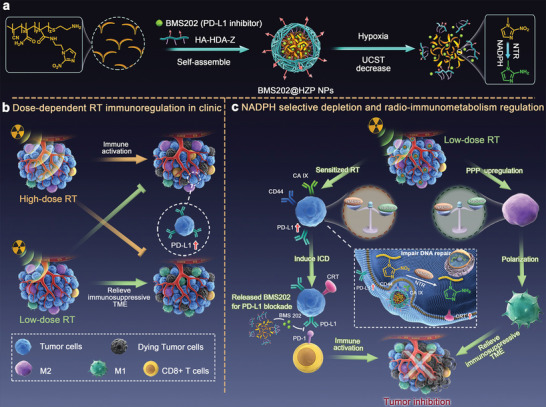
Production and application of the BMS202@HZP NPs. a) Schematic illustration of design and preparation of BMS202@HZP NPs. b) Dose‐dependent RT immunoregulation in clinic. c) Mechanism of BMS202@HZP NPs‐mediated NADPH selective depletion and radio‐immunometabolism regulation for synergistically anti‐PDL1 therapy against TNBC.

Due to the significant impacts of radiotherapy (RT) on TME and systemic immunity, the combination of RT and anti‐PD‐L1 therapy provides a promising strategy to treat TNBC. On the one hand, high‐dose RT would pose damages to the DNA of cancer cells, which can not only induce immunogenic cell death (ICD), but also activate the ATM/ATR/Chk1 signaling pathway to upregulate PD‐L1 expression, eventually reversing the “cold tumor” to “hot tumor” and increasing the anti‐PD‐L1 therapy response.^[^ ^6^ ^]^ On the other hand, low‐dose RT (LDRT, 0.5–2 Gy) would remodel the TME and alleviate its immunosuppression through regulating the immunometabolism of TME (**Figure** **1**).^[^ ^7^ ^]^ The radiation dosage employed during RT is closely related to its immunomodulatory effectiveness,^[^ ^8^ ^]^ where it is challenging to simultaneously increase the expression of PD‐L1 in tumor cells and relieve the immunosuppression of TME through RT alone with a fixed‐dosage. One of the key factors for this dilemma is the existence of the antioxidant compounds in the tumor,^[^ ^9^ ^]^ such as the reduced nicotinamide adenine dinucleotide phosphate (NADPH). Responding to the enhanced oxidative stress induced by RT, the pentose phosphate pathways (PPP) of the whole tumor tissue would be upregulated, followed by enhanced production of NADPH.^[^ ^10^ ^]^ When NADPH is over‐generated in immune cells, such as the tumor‐associated macrophages (TAMs), it would facilitate the polarization of immune cells toward M1‐like phenotype.^[^ ^11^ ^]^ Beyond, NADPH that drives many metabolic alterations would also participate in the synthesis of fatty acid and plasma membrane in newly activated CD8+ T cells and M1 macrophages,^[^ ^12^ ^]^ which can contribute to the modulation of immunometabolism and the relief of immunosuppression of TME. However, when the level of NADPH inside tumor cells is elevated, it can turn into a building block of plasma membrane in rapidly growing tumor cells, which would assist the repairment of the damaged DNA in tumor cells and inhibit their immunogenic death and PD‐L1 upregulation.^[^ ^13^ ^]^ Therefore, selective depletion of NADPH in tumor cells is the key to solve the puzzle of RT‐based treatments of TNBC.

In order to reduce the intratumoral antioxidant compounds for RT sensitization, in addition to PPP inhibitors, a few functional nanomaterials have been developed to not only function as vehicles for drug delivery and controlled release, but to possess the capacity of scavenging the antioxidant compounds.^[^ ^9^, ^10^ ^]^ Therefore, it is urgent to develop an effective “drug‐like” adjuvant nanomaterial to selectively deplete the tumor cellular NADPH and immobilize the DNA damage during LDRT to increase the expression of PD‐L1, while simultaneously alleviating the immunosuppression of TME by maintaining NADPH at a high level in immune cells, eventually improving the immune response of anti‐PD‐L1 therapy plus LDRT against TNBC. Herein, a multifunctional “drug‐like” copolymer with the auto‐changeable upper critical solution temperature (UCST), named poly(acrylonitrile‐*co*‐acrylamide‐*co*‐*N*‐(2‐(2‐nitro‐1*H*‐imidazol‐1‐yl)ethyl)acrylamide (PAAN), was synthesized and exploited to prepare nanoparticles (NPs) for delivering BMS202 (an antagonist of PD‐1/PD‐L1 interaction) and facilitate the depletion of NADPH in cancer cells, which was beneficial for LDRT to regulate the immunometabolism of TME and reinforce the anti‐PD‐L1 therapy against TNBC (Figure 1). Briefly, this type of nanomedicine, BMS202@HZP NPs, could be prone to accumulate in TNBC cells by virtue of its dual‐targeting ability to both CD44 receptors and carbonic anhydrase IX (CA IX) on cell membrane. Meanwhile, with the assistance of nitroreductase (NTR) over‐expressed in cancer cells under hypoxia, the NADPH inside cancer cells could be scavenged by the nitroimidazole groups in PAAN copolymer to sensitize LDRT, immobilize DNA damage of cancer cells, and facilitate their PD‐L1 expression and immunogenic death. Contrastly, due to lack of NTR in TAMs, the elevated NADPH in TAMs would be not depleted, which could facilitate their polarizations toward M1‐like phenotypes and relieve the immunosuppression of TME. Additionally, once the nitroimidazole groups in this nanomedicine were reduced to the hydrophilic aminoimidazole groups by NADPH in tumor cells, the UCST of PAAN copolymer could be automatically decreased to the point below the physiological temperature by alternating the hydrophilicity to hydrophobicity ratio, causing the rapid disintegration of this nanomedicine and on‐demand release of the PD‐L1 inhibitor BMS202. In comparison with the preclinical protocol (24 Gy in 3 fractions) and previous studies,^[^ ^6^, ^10^, ^14^ ^]^ the significantly lower dose RT (6 Gy in 3 fractions) in our study simultaneously achieved the reconstruction of the tumor immuno‐microenvironment and the reinforcement of anti‐PD‐L1 therapy while further avoiding the adverse effects, because of the above advantages of the BMS202@HZP NPs (Figure 1a,c). Generally, if traditional nanomedicines are regarded as flowering plants, the encapsulated drugs would be like “flowers” to initiate the therapeutic efficacy, while the carrier materials would act as “green leaves” to augment their treatment effectiveness. In sharp contrast, our novel nanocarriers could not only serve as the “green leaves” for drug delivery and controlled release, but also play a unique role as the “flowers” for specific clearance of NADPH in boosting the therapy against TNBC via metabolic immunoregulation, which provides an alternative strategy for combining RT with anti‐PDL1 therapy against TNBC.

## 2 Results and Discussion

### 2.1 Preparation and Characterization of BMS202@HZP NPs

The auto‐changeable UCST polymer PAAN was synthesized through the radical polymerization of acrylonitrile (AN), acrylamide (AAm), and *N*‐(2‐(2‐nitro‐1*H*‐imidazol‐1‐yl)ethyl)acrylamide (NIEAAm) (Figure S1a, Supporting Information), as the carrier materials for encapsulating BMS202 and its subsequent controlled release. Meanwhile, a lipoid (i.e., hyaluronic acid‐hexadecylamine‐ acetazolamide, HA‐HDA‐Z) was obtained by introducing acetazolamide (Z) and hexadecylamine (HDA) groups into hyaluronic acid (HA) in succession (Figure S1b, Supporting Information), as the emulsifier for formulating the PAAN NPs with a shell of lipoid via hexadecyl anchors. The chemical structures of PAAN and HA‐HDA‐Z were confirmed by ^1^H NMR (Figure S2a,b, Supporting Information). The content of nitroimidazole groups in PAAN polymer was 261 µg per mg of polymer according to UV‐vis spectrum analysis of NIEAAm and PAAN polymer in dimethyl sulfoxide (Figure S3, Supporting Information). The electrochemical test results of cyclic voltammogram revealed that PAAN polymer possessed similar electron affinity with half‐wave potential *E*_1/2_ = −0.660 V to NIEAAm with *E*_1/2_ = −1.049 V (Figure S4, Supporting Information), suggesting the nitroimidazole groups in PAAN polymer still showed the efficient electron affinity. Due to the electron affinity, the nitroimidazole groups in PAAN polymer could be reduced by NADPH under hypoxic condition containing NTR, which was verified by the reduced absorbance intensity of nitroimidazole group at 330 nm after incubating PAAN in the presence of NADPH and NTR under hypoxia for 24 h. However, addition of a single reductant (e.g., GSH or NADPH) into the solution of PAAN induced a negligible change or a slight decrease (Figure S5, Supporting Information). These results confirmed that the PAAN polymer could react with NADPH and deplete it with assistance of NTR, impairing the DNA damage repair to sensitize RT even under hypoxia.

**Figure 2:**
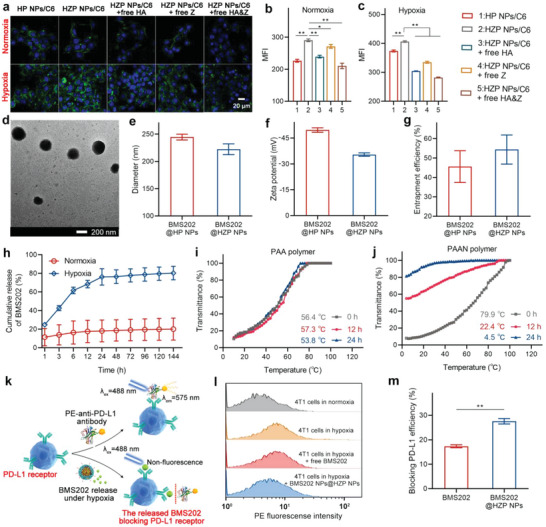
Targeting capacity, structure, payload release profiles, and blocking PD‐L1 receptor efficacy of NPs. a) CLSM images and b,c) flow cytometry analysis of 4T1 cells incubated with HP NPs/C6 and HZP NPs/C6 with or without pretreatment of HA ([HA]  =  10 mg mL^−1^) and Z ([Z]   = 0.2 mg mL^−1^). The blue color represents nucleus and green color represents C6‐loaded NPs. d) Morphology of BMS202@HZP NPs. e–g) Characterization of different NPs, including e) diameter, f) zeta potential, and g) encapsulation efficiency. h) Cumulative release of BMS202 from BMS202@HZP NPs incubated in PBS (pH 7.4, 0.01 m) containing NADPH and NTR under normoxia and hypoxia at 37 °C, respectively. i,j) Turbidity heating profiles of i) the PAA polymer and j) PAAN polymer after incubation with NADPH and NTR at 37 °C under argon protection for 0, 12, and 24 h. k) Schematic diagram of the BMS202 released from BMS202@HZP NPs to inhibit the interaction between PE‐anti‐PD‐L1 antibody and PD‐L1 receptor under hypoxia. l) PE fluorescence intensities of anti‐PD‐L1 antibodies in 4T1 cells treated with or without different drug formulations under normoxia and hypoxia. m) Blocking PD‐L1 efficiency of BMS202 and BMS202@HZP NPs in hypoxia (mean ± SD, *n* = 3, *p < 0.05, **p < 0.01).

The blank NPs without BMS202 loading, denoted as HZP NPs, were prepared using HA‐HDA‐Z and PAAN polymers, which exhibited spherical shapes with an average diameter of 210 nm and *ζ*‐potential of −31.2 mV (Figure S6a–c, Supporting Information). Anionic HA, present on the surface of HZP NPs, played a critical role in promoting the stability of HZP NPs in a simulated physiological condition, reflected as the negligible change of NPs’ hydrodynamic diameters during 48 h of incubation (Figure S6d, Supporting Information). The targeting capacity of HZP NPs on 4T1 cells under normoxia (21% O_2_) and hypoxia (1% O_2_) was evaluated using flow cytometry and confocal laser scanning microscopy (CLSM) with coumarin 6 (C6) as a fluorescent probe and CD44 single‐targeting HP NPs as control. It has been reported that CA IX receptors are often over‐expressed in cancer cells under hypoxia and CD44 is a cell surface receptor indicating malignancy.^[^ ^15^ ^]^ Thus, we hypothesized that the HZP NPs could efficiently interact with 4T1 cells under hypoxia that benefited from the specific binding of HA and Z to CD44 and CA IX receptors, respectively. Compared to the HP NPs/C6 lacking Z modification, more HZP NPs/C6 accumulated in 4T1 cells and highlighted its advantages in targeting cancer cells under hypoxia (**Figure** **2**a–c). Moreover, in the competitive inhibition experiments, the amount of HZP NPs/C6 existing in 4T1 cells was significantly reduced because of the preblockade of CD44 and CA IX by free HA and Z, further confirming their targeting ability to 4T1 cells under hypoxia. Furthermore, to further demonstrate the high selectivity of HZP NPs to 4T1 cells under hypoxia, we incubated HZP NPs/C6 with 4T1 cells, RAW264.7 macrophages, and T cells under normoxia and hypoxia for 4 h, respectively. As shown in Figure S7a–d in the Supporting Information, compared to T cells and RAW264.7 macrophages, more HZP NPs/C6 accumulated in 4T1 cells. Especially under hypoxia, the intracellular fluorescence intensity of 4T1 cells was 2.4‐ and 2.3‐fold higher than that of T cells and RAW264.7 macrophages, indicating that HZP NPs were prone to target TNBC cells.

After loading the antagonist of PD‐1/PD‐L1 interactions (BMS202), the as‐obtained spherical BMS202@HZP NPs presented the similar morphological characteristics (222.2 nm, −35.4 mV) to the blank HZP NPs (Figure 2d–f), and the encapsulation efficiency of BMS202 for BMS202@HZP NPs was 54.5% (Figure 2g). The release profiles of BMS202 from BMS202@HZP NPs under various conditions were measured to evaluate the responsiveness of PAAN core with auto‐changeable UCST property under hypoxia in presentence of NADPH and NTR. Under normoxia, only 20.1% of BMS202 was released after 144 h incubation of BMS202@HZP NPs at pH 7.4 (Figure 2h). In contrast, the release of up to 61.6% and 80.3% BMS202 was observed for BMS202@HZP NPs incubated under hypoxia in presentence of NADPH and NTR for 6 and 144 h, respectively, revealing that the HZP NPs achieved the effectively controlled drug release. As reported in our previous studies, the hydrophobicity acted an important role in thermo‐responsiveness of UCST polymers.^[^ ^16^ ^]^ When the ratio of hydrophilicity versus hydrophobicity of UCST polymers increased, the intermolecular forces among polymers would be weakened, leading to a decreased UCST. As above demonstrated, the hydrophobic nitroimidazole groups in the PAAN chains could transform into hydrophilic aminoimidazole groups under hypoxia in the presence of NADPH and NTR,^[^ ^17^ ^]^ which should be the main reason for realizing its auto‐changeable UCST. Thus, compared to PAA polymer without nitroimidazole group (Figure 2i), the UCST of PAAN polymer significantly decreased from 79.9 to 22.4 and 4.5 °C after 12 and 24 h of incubation under hypoxic condition containing NADPH and NTR (Figure 2j), respectively, demonstrating that the UCST of PAAN polymer could be auto‐changed under hypoxia in the presence of NADPH and NTR. By virtue of this advantage of PAAN as carrier, HZP NPs would avoid several adverse side effects caused by the undesired leakage of BMS202 during systemic circulation. Once entering the tumor under hypoxia, BMS202@HZP NPs would collapse rapidly to release BMS202. More importantly, when 4T1 cells were treated with BMS202@HZP NPs under hypoxia, the released BMS202 from BMS202@HZP NPs would specifically bind to PD‐L1 receptors, potentially inhibiting the PD‐1/PD‐L1 interaction to realize ICB therapy. The high binding capacity of BMS202 to PD‐L1 was observed, as shown by a reduced binding efficiency of PE‐anti‐PD‐L1 antibody to PD‐L1 receptors for the cells pretreated with BMS202@HZP NPs, revealing a good potentiality of anti‐PD‐L1 ICB therapy of BMS202@HZP NPs (Figure 2k–m).

To further demonstrate that the imidazole groups in PAAN were prone to selectively scavenge the NADPH inside cancer cells, the RAW264.7 macrophages and 4T1 cells were radiated with 2 Gy of X‐ray, and then treated with 0.9% NaCl, blank HZP NPs, and BMS202@HZP NPs, respectively. To adapt to the enhanced oxidative stress induced by RT, the intracellular NADPH/NADP^+^ ratios of both macrophages and 4T1 cells were obviously increased (Figure S8, Supporting Information). Moreover, when the RAW264.7 macrophages were treated with the blank HZP NPs and BMS202@HZP NPs, the NADPH/NADP^+^ ratio still stayed at the high level compared to the macrophages without any treatments (Figure S8a, Supporting Information). However, the NADPH/NADP^+^ ratio of 4T1 cells would obviously decrease under both normoxia (Figure S8b, Supporting Information) and hypoxia (Figure S8c, Supporting Information) once co‐culturing the blank HZP NPs and BMS202@HZP NPs with 4T1 cells together. Thus, it was demonstrated that the BMS202@HZP NPs could scavenge the NADPH inside 4T1 cells, which was attributable to the targeting capacity of HZP NPs to 4T1 cells as well as the overexpression of NTR in cancer cells.^[^ ^18^ ^]^

### 2.2 In Vitro Antitumor Efficacy of BMS202@HZP NPs

**Figure 3:**
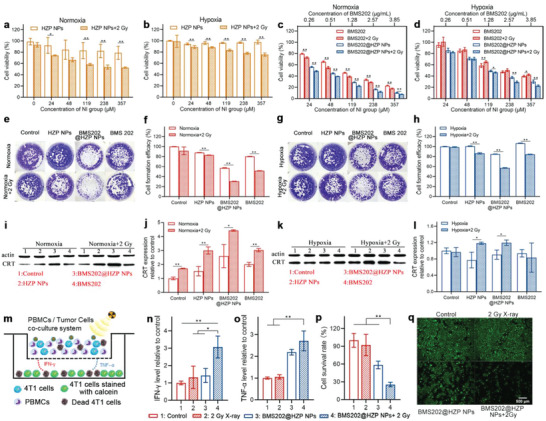
In vitro therapeutic and immunoregulatory effects of BMS202@HZP NPs. a–d) Cytotoxicity evaluation of blank a,b) HZP NPs, c,d) BMS202 and BMS202@HZP NPs with or without LDRT (2 Gy) against 4T1 cells at various NI‐equivalent concentrations under hypoxia and normoxia (*n* = 5). e–l) Representative images of e,g) colony formation assay, f,h) clone formation efficacy, i,k) CRT expression, and j,l) western blot analysis of 4T1 cells treated with free BMS202, blank HZP NPs, and BMS202@HZP NPs‐mediated 2 Gy X‐ray radiation (nitroimidazole [NI]  =  238 × 10^−6^ m, [BMS202] = 2.57 µg mL^−1^) under hypoxia or normoxia (*n* = 3). m) Schematic diagram of the experimental methodology for distant tumor cell treatment in vitro using a co‐culture model in the transwell system. n,o) The levels of n) IFN‐*γ* and o) TNF‐*α* in cell supernatants collected from the lower chamber (*n* = 3). p) Cell survival rate and q) image of distant 4T1 cells stained with calcein‐AM in the lower chamber (*n* = 3) (mean ± SD, *p < 0.05, **p < 0.01).

The nitroimidazole groups in PAAN polymer could deplete NADPH and immobilize DNA damage during RT due to their higher electron affinity, contributing to an increased in vitro antitumor efficacy. To prove this, we evaluated the cytotoxicity of BMS202@HZP NPs against 4T1 cells upon the low‐dose (2 Gy) X‐ray radiation using 3‐(4, 5‐dimethylthiazolyl‐2)‐2, 5‐diphenyltetrazolium bromide assay. Indeed, the viability of 4T1 cells gradually decreased with an increase of nitroimidazole concentration after the blank HZP NPs‐mediated LDRT, regardless of the oxygen level (**Figure** **3**a,b). Meanwhile, since BMS202 can not only inhibit the formation of PD‐1/PD‐L1 complex but also induce cytotoxicity to cancer cells, BMS202@HZP NPs showed the enhanced inhibitory on 4T1 cells growth (Figure 3c,d). Additionally, the combination indexes of BMS202 and nitroimidazole‐sensitized LDRT at IC_50_ values (Table S1, Supporting Information) under normoxia (0.37) and hypoxia (0.42) were much lower than 1 for the BMS202@HZP NPs‐mediated LDRT, indicating a good synergism mediated by BMS202@HZP NPs. Then, the long‐term radiation‐sensitization effect of BMS202@HZP NPs was also examined on 4T1 cells using clone formation assay. Once 4T1 cells were treated with BMS202@HZP NPs plus LDRT, there were only around 30.8% and 57.1% clones formed under normoxia (Figure 3e,f) and hypoxia (Figure 3g,h), respectively, which were much less for the 4T1 cells undergoing the treatments of free BMS202 and blank HZP NPs‐mediated LDRT. Collectively, BMS202@HZP NPs‐mediated LDRT could suppress the proliferation of 4T1 cells, potentially implying their capacity of scavenging the tumor intracellular NADPH to sensitize LDRT.

In addition to enhancing the LDRT efficiency, the purpose of incorporating nitroimidazole groups into BMS202@HZP NPs is to realize the efficient ICD and activate the immune response against TNBC, by virtue of depleting NADPH inside tumor cells to impair the DNA repair during LDRT. Besides, the cytotoxic BMS202 could also make contributions to inducing ICD. Calreticulin (CRT) is one of major hallmarks of ICD, which is a well‐known “eat‐me” signal for dendritic cells (DCs) uptake and activating immune system.^[^ ^19^ ^]^ Thus, the expressions of CRT on 4T1 cells with different treatments were first measured using western blot. It was found that the CRT expressions of 4T1 cells after the treatments of HZP NPs or BMS202@HZP NPs‐mediated LDRT were upregulated (Figure 3i–l), especially for the treatment of BMS202@HZP NPs plus LDRT, which increased by 4.4‐fold and 1.2‐fold compared to the untreated 4T1 cells under normoxia and hypoxia, respectively. As such, BMS202@HZP NPs‐mediated LDRT could effectively induce TNBC immunogenic death.

To further evaluate activities of immune cells against metastatic cells after BMS202@HZP NPs‐mediated LDRT, a double‐layered cell model was established using a transwell system as illustrated in Figure 3m. Briefly, the peripheral blood mononuclear cells (PBMCs) and 4T1 cells were co‐cultured in upper wells under hypoxia and performed with different treatments, then the level of several cytokines that promoted immune responses in lower wells (preseeded with 4T1 cells) was measured. Meanwhile, the viability of tumor cells in lower wells was determined via calcein‐AM staining. Due to the hypoxia, LDRT alone hardly generated radicals to induce the ICD and facilitated PBMCs to secrete IFN‐*γ* and TNF‐*α*, causing the negligible inhibitory effect on the proliferation of metastatic 4T1 cells (Figure 3n,o). In sharp contrast, the enhanced content of IFN‐*γ* and TNF‐*α* were minored in the groups of BMS202@HZP NPs with or without LDRT, further inducing the 4T1 cells death in lower wells. Especially, BMS202@HZP NPs‐mediated LDRT presented the most significant killing effect against 4T1 cells and reflected as only 25.4% of viable cells stained by calcein‐AM in lower cells, which were 3.6‐fold and 2.3‐fold lower than the 4T1 cells that only received LDRT or BMS202@HZP NPs treatments (Figure 3p,q). This phenomenon could be explained by the following factors: for one thing, BMS202 could relieve PD‐1/PD‐L1 immunosuppressive pathway to boost T cells response and facilitate cytokines production; for another thing, due to the existence of nitroimidazole in HZP NPs, BMS202@HZP NPs were prone to scavenge the NADPH inside the 4T1 cells to impair the DNA damage repair and induce immunogenic death of 4T1 cells under hypoxia, which would also activate PBMCs to release more cytotoxic cytokines against metastatic 4T1 cells.

### 2.3 In Vivo Biodistribution of BMS202@HZP NPs

The effective delivery of nanomedicine to tumor is the prerequisite to achieving better therapeutic efficacy in vivo. However, there are some factors, such as the dense blood vessels and elevated interstitial fluid pressure, limiting the intratumoral delivery of nanomedicine. The preclinical data implied that only 0.7% of injected dose of NPs accumulated in human tumors.^[^ ^20^ ^]^ To address this challenge, besides modifying the surface of NPs with targeted ligands, several exogenous stimuli (e.g., radiation and heat) are also effective in promoting accumulation of NPs in tumors.^[^ ^21^ ^]^ According to the previous studies, X‐ray radiation could alter the fluid dynamics of tumor blood vessels and enhance the accumulation of NPs in tumors.^[^ ^21^ ^]^ In this study, DiR (a near‐infrared fluorescence probe)‐loaded HZP NPs could indeed better accumulate in tumors pretreated with X‐ray radiation (Figure S9, Supporting Information), presenting 2.5‐fold, 1.5‐fold, and 1.1‐fold enhancement in tumor fluorescence intensity as compared to the group of free DiR, DiR@HP NPs, and DiR@HZP NPs without the pretreatment of X‐ray radiation, respectively. To further confirm whether the increased vascular permeability was caused by X‐ray radiation, the mice pretreated with or without X‐ray radiation were i.v. injected the Evans blue dye at 24 h post‐injection of 0.9% NaCl, BMS202@HP NPs, or BMS202@HZP NPs. After measuring the content of extravasated Evans blue in tumors, we found that there was no significant difference in intratumoral Evans blue level among the groups of 0.9% NaCl, BMS202@HP NPs, and BMS202@HZP NPs. Excitingly, there was 1.6–1.7‐fold enhancement on Evans blue dye extravasation for the group of BMS202@HZP NPs plus LDRT in comparison to the other groups (Figure S10, Supporting Information). These results further demonstrated that the X‐ray radiation could serve as an enhancer for augmenting enhanced permeability and retention effect. Besides, CD44/CA IX dual‐targeted DiR@HZP NPs presented higher fluorescence intensity than the single‐targeted DiR@HZP NPs, verifying that the specific interaction between HA/CD44 receptor and Z/CA IX receptor could promote HZP NPs to target hypoxic 4T1 tumor. Finally, both X‐ray radiation and HA/Z modification jointly improved the accumulation of therapeutics at tumor sites, which would lay the foundation for effective TNBC therapy.

### 2.4 In Vivo Therapeutic Efficacy of BMS202@HZP NPs

**Figure 4:**
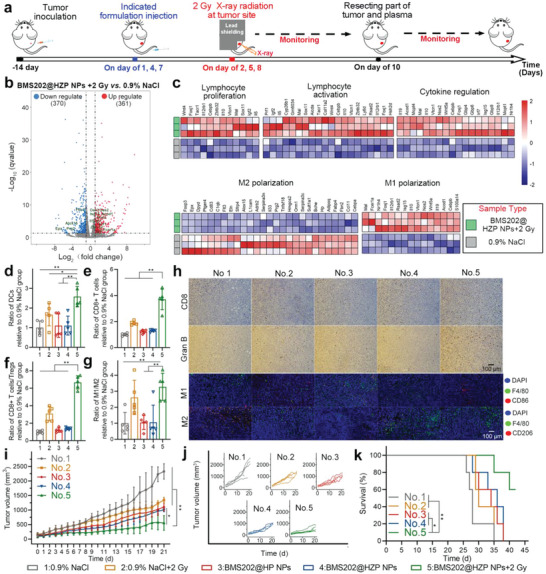
BMS202@HZP NPs plus LDRT relieving the immunosuppression of TME to achieve the enhanced antitumor efficacy. a) Treatment schedule for BMS202@HZP NPs‐mediated LDRT against subcutaneous 4T1 tumor on Balb/c mice. b) Volcano plot for displaying genes expression in tumor. Red dots represent upregulated genes, gray dots represent not differentially expressed genes, and blue dots represent downregulated genes in BMS202@HZP NPs‐mediated LDRT group versus 0.9% NaCl group (cut off: fold change ≥ 2, FDR‐adjusted *p‐*value < 0.05). c) Heatmap of differentially expressed genes associated with immune response of 4T1 tumors following the indicated treatments on day 10 (fold change ≥ 2, FDR‐adjusted *p‐*value < 0.05). d) DCs, e) CD8+ T cells, f) CD8+ T cells/T_regs_ ratio, and g) M1/M2 ratio in 4T1 tumors following the indicated treatments on day 10 (*n* = 5). h) IHC staining for CD8 and Gran B, and immunofluorescence staining for CD86 (hallmark of M1 phenotype, red), F4/80 (hallmark of TAM, green), CD206 (hallmark of M2 phenotype, red), and nucleuses (blue) of tumor tissue in 4T1 tumors following the indicated treatments on day 10. i) Primary tumor growth and j) their spaghetti curves during experiment (*n* = 5). k) Survival curves of mice‐bearing 4T1 tumors after tumor inoculation (*n* = 5, log‐rank test) (mean ± SD, *p < 0.05, **p < 0.01).

RT was reported to facilitate the PD‐L1 upregulation,^[^ ^22^ ^]^ which also has been verified in our study with results shown in Figure S11 in the Supporting Information. Compared to the LDRT (2 Gy) alone, the BMS202@HZP NPs‐mediated LDRT resulted in the enhanced PD‐L1 expression in 4T1 tumor (Figure S11, Supporting Information). As mentioned above, the low or negative expression of PD‐L1 was one of the major reasons for the low response rate of anti‐PD‐L1 therapy among TNBC patients. Thus, promoting the PD‐L1 expression by BMS202@HZP NPs‐mediated LDRT has the potential to increase the response rate and efficiency of anti‐PD‐L1 therapy against TNBC, facilitate the CD8+ T cells recruitment, and relieve the immunosuppression of TME. To prove this, the mice bearing a single 4T1 subcutaneous tumor were subjected to 0.9% NaCl, BMS202@HP NPs, and BMS@HZP NPs with or without LDRT (2 Gy) when the tumor volume reached around 150 mm^3^, and part of tumors and plasma were harvested to evaluate the immune responses on 10 days as illustrated in **Figure** **4**a. Additionally, the transcriptome sequencing and analysis of total RNA were performed. BMS202@HZP NPs‐mediated LDRT induced the significant changes in 731 genes in tumor in comparison to 0.9% NaCl treatment (Figure 4b). Thereinto, there were 361 genes upregulated and 370 downregulated. Moreover, through gene ontology analysis, it is shown that among the differentially expressed genes, 61 genes were associated with immune system. As shown in Figure 4b,c, BMS202@HZP NPs‐mediated LDRT caused the upregulation of the genes correlated with the proliferation and activation of T cells (e.g., Igf2, Zbtb32, Il12rb1, and Tacr1), the interferon response genes (e.g., Oas2 and Oas3) for effector T‐cell recognition of tumor cells and the genes polarized TAMs into immunostimulatory M1 phenotype (e.g., Nos2, Acod1, Isg15, and Fcer1a). Meanwhile, the expression of genes relative to the process of polarizing TAMs into an immunostimulatory M2 phenotype (e.g., Epx, Prg2, Alox15, Fbn2) reduced obviously after the treatment of BMS202@HZP NPs plus LDRT. Accordingly, it was indicated that BMS202@HZP NPs‐mediated LDRT possessed the potential to increase antitumor immune responses and relieve the immunosuppression of TME.

On account of LDRT capacity to regulate immune response, there was a slight increase in the amount of CD3+CD8+ T cells and matured DCs in tumors received 2 Gy of radiation in comparison to 0.9% NaCl‐treated group (Figure 4d,e), and the ratios of CD8+ T cells versus regulator T cells (T_reg_) and M1 versus M2 also increased (Figure 4f,g). However, the ratios of various immune cells were not obviously altered for the mice administrated with BMS202@HP NPs or BMS202@HZP NPs in the absence of low‐dose X‐ray radiation (2 Gy). Moreover, BMS202@HZP NPs‐mediated LDRT could further strengthen the immune activities in vitro, and thus this treatment achieved the maximal elevation of the CD8+ T cells infiltration (Figure 4e,h), the DCs maturation (Figure 4d), and the Granzyme B (Gran B) production (Figure 4h) in tumor among various groups. Meanwhile, there were more M1‐like phenotype macrophages and less M2‐like phenotype macrophages detected in tumor after BMS202@HZP NPs‐mediated LDRT. The ratio of M1*/*M2 in this group was 3.3‐, 1.3‐, 3.1‐, and 3.1‐fold higher than that in groups of 0.9% NaCl, 2 Gy radiation, BMS202@HP NPs, and BMS202@HZP NPs, respectively (Figure 4g). Immunofluorescence staining for CD86 (hallmark of M1) and CD206 (hallmark of M2) also presented the consistent results (Figure 4h). Considering TAMs account for a portion even up to 50% in tumor mass and represent the major population of immune cells in TME,^[^ ^23^ ^]^ BMS202@HZP NPs‐mediated LDRT could polarize the TAMs into M1‐phenotype and relieve the immunosuppression of TME.

The above results confirmed that BMS202@HZP NPs‐mediated LDRT could activate immune response and reprogram TME toward immunostimulatory phenotype, so as to enhance the capacity for inhibiting tumor growth. As expected, there was an obvious improvement in suppressing tumor growth (Figure 4i,j and Figure S12, Supporting Information) and prolonging survival rate (Figure 4k) for the mice received BMS202@HZP NPs‐mediated LDRT, and its tumor inhibitory efficiency was 76.2% at the end of experiment (Figure S13, Supporting Information). In contrast, the treatment of 2 Gy radiation alone and nanomedicines (BMS202@HP NPs or BMS202@HZP NPs) in absence of LDRT just moderately suppressed the growth of 4T1 tumors. Overall, BMS202@HZP NPs‐mediated LDRT could achieve an excellent capacity of eliminating tumor tissue.

### 2.5 Mechanisms of Immune Regulation Analysis based on Metabolomics

**Figure 5:**
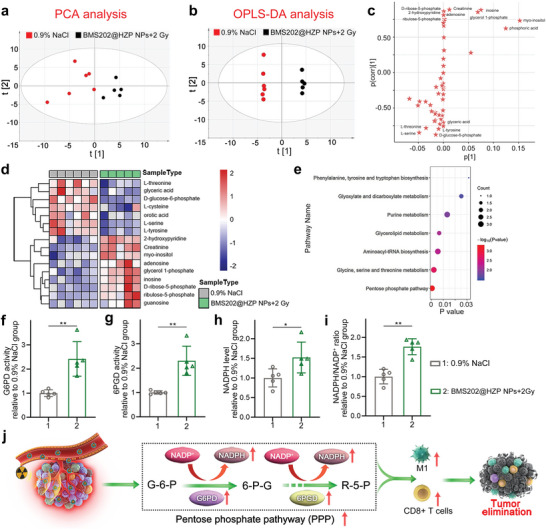
Analysis of metabolites in tumor tissue treated with BMS202@HZP NPs plus LDRT. a) PCA score plot and b) OPLS‐DA score plot for 0.9% NaCl (red dots) and BMS202@HZP NPs‐mediated LDRT groups (black dots). c) S‐plot of OPLS‐DA model. d) Heatmap of differential metabolites of tumor in BMS202@HZP NPs‐mediated LDRT compared to 0.9% NaCl group. e) Metabolic pathway enrichment analysis based on differential metabolites. f–i) Enzyme activity of f) G6PD and g) 6PGD, h) level of NADPH, and i) ratio of NADPH/NADP^+^ in tumor tissue after different treatments (mean ± SD, *n* = 5, *p < 0.05, **p < 0.01). j) Schematic illustration of the proposed mechanism for BMS202@HZP NPs‐mediated LDRT to reverse relieve suppression of TME by upregulating PPP.

Metabolomics is a state‐of‐the‐art tool to identify and quantify the downstream small molecules present in biological systems, aiming to gain insight into the system's metabolic state.^[^ ^24^ ^]^ More importantly, the metabolism of both cancer cells and immune cells is believed to determine their fate and relevant TME.^[^ ^12a,d^ ^]^ Therefore, we measured and analyzed the metabolisms of the tumors treated by BMS202@HZP NPs‐mediated LDRT using gas chromatography‐mass spectrometry, with the purpose of elucidating the synergism mechanisms behind anti‐PD‐L1 therapy and LDRT. In this study, a total of 52 metabolites were identified in tumors for the groups of BMS202@HZP NPs‐mediated LDRT and 0.9% NaCl, which are shown in Table S2 in the Supporting Information. The principal component analysis (PCA) was then performed to produce an unbiased overview of the metabolic datasets, which exhibited a clear and separated clustering trend of metabolic datasets between these two groups (**Figure** **5**a). Furthermore, the supervised orthogonal partial least‐squares discrimination analysis (OPLS‐DA) was employed to optimize the group discrimination model. As shown in Figure 5b, the score plots of BMS202@HZP NPs‐mediated LDRT group were far from those of the 0.9% NaCl group, which revealed that the metabolic profiles in tumor significantly changed after the synergistic therapy. It is worth mentioning that the cumulative R2 and Q2 values of OPLS‐DA were 0.860 and 0.800, respectively, verifying the good qualities of this model. As exhibited in S‐plot of OPLS‐DA (Figure 5c), the variable importance for the projection (VIP) values of labeled metabolites (14 compounds) was greater than 1, suggesting these compounds might be altered metabolites between these two groups. Besides, the heatmap clustering analysis of the tumor differential metabolites between 0.9% NaCl and BMS202@HZP NPs‐mediated LDRT groups was also performed, and a total of 16 differential metabolites (false discovery rate (FDR)‐adjusted *p‐*value <0.05) were screened out and are shown in Figure 5d. Based on these results, the significantly differential metabolites, matching the condition of both VIP > 1 and FDR‐adjusted *p‐*value < 0.05, were identified, which revealed that BMS202@HZP NPs‐mediated LDRT induced the significant elevation of adenosine, D‐ribose‐5‐phosphate, glycerol 1‐phosphate, ribulose‐5‐phosphate, etc., and decline of D‐glucose‐6‐phosphate, glyceric acid, etc. (Table S3, Supporting Information).

The pathway enrichment analysis based on significantly differential metabolites was then conducted, and seven metabolic pathways were significantly altered in BMS202@HZP NPs‐mediated LDRT group. Among them, the most significant difference occurred in PPP (Figure 5e); thus, the activity and level of enzymes and compounds associated with PPP were measured. The activities of glucose‐6‐phosphate dehydrogenase (G6PD) and 6‐phosphogluconate dehydrogenase (6PGD) for BMS202@HZP NPs‐mediated LDRT were increased by 2.4‐ and 2.3‐fold as compared with 0.9% NaCl group, respectively (Figure 5f,g). In agreement with enzymatic activity levels of G6PD and 6PGD, there were also 1.5‐fold and 1.8‐fold enhancements on the level of NADPH and the ratio of NADPH/NADP^+^ for the synergistically therapeutic group, respectively (Figure 5h,i). Since BMS202@HZP NPs could selectively target 4T1 cells and scavenge their intracellular NADPH by nitroimidazole group, these results suggested the PPP of TME was further elevated to produce more NADPH after this therapy. As discussed above, the PPP, as one of the important specific glycolytic mediators, influences the division and activation of immune cells.^[^ ^12a,b^ ^]^ NADPH generated by PPP is a molecule with essential roles in the synthesis of biomolecules, like fatty acid synthesis, which is the primary source for fatty acid and plasma membrane synthesis in newly activated CD8+ T cells.^[^ ^12b,d^ ^]^ Furthermore, NADPH is the crucial substrate of the NADPH oxidase (NOX) pathway, which is exploited by activated macrophages to kill cancer cells.^[^ ^25^ ^]^ Thus, the engagement of the PPP is a peculiar feature of activated CD8+ T cells and M1. Besides, since NADPH also plays the key role in maintaining cellular redox, the upregulated NADPH could facilitate the polarization of TAMs from M2‐like phenotype toward M1‐like phenotype upon scavenging reactive oxygen species.^[^ ^11^ ^]^ Therefore, the elevated PPP of TME during BMS202@HZP NPs‐mediated LDRT should be responsible for relieving the immunosuppression of TME, serving as an important proinflammatory programmer of antitumor response (Figure 5j).

### 2.6 Abscopal Effect and Lung Metastasis Treatment

**Figure 6:**
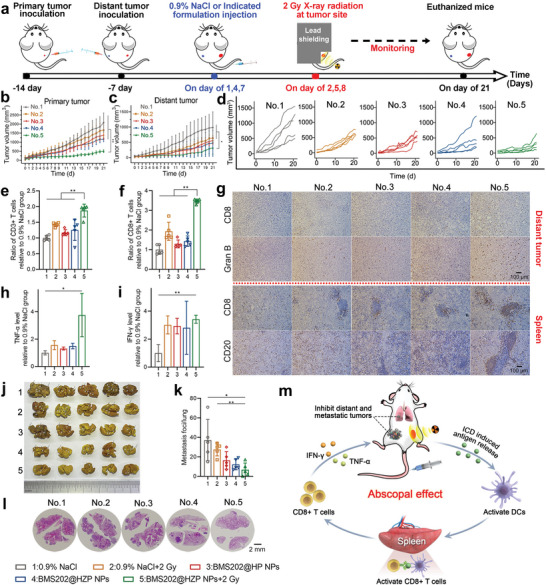
Abscopal effects and systemic antitumor immunity induced by BMS202@HZP NPs‐mediated LDRT. a) Treatment schedule for BMS202@HZP NPs‐mediated LDRT against primary and distant 4T1 tumors. b) Primary tumor growth, c) distant tumor growth, and d) spaghetti curves of distant tumors during experiments. e–i) The level of e) CD3+ T cells and f) CD8+ T cells in distant tumor tissue, and the level of h) TNF‐*α* and i) IFN‐*γ* in plasma (*n* = 3) following the indicated treatments on day 21. g) IHC staining of CD8+ T cells and Gran B of distant tumor, and CD20 and CD8 of spleen following the indicated treatments on day 21. j) Morphology, k) number, and l) H&E staining images of lung metastatic nodules. m) Schematic diagram of BMS202@HZP NPs‐mediated LDRT inducing strong antitumor immunological responses to reinforce the abscopal effect against distant tumor growth and tumor metastasis (mean ± SD, *n* = 5, *p < 0.05, **p < 0.01).

To evaluate abscopal effects of BMS202@HZP NPs‐mediated LDRT, the 4T1 bilateral tumor model was established and various treatments were performed, as illustrated in **Figure** **6**a. It was found that the growth profiles of primary tumors on bilateral tumor model were consistent with those on unilateral tumor model, where the optimal tumor‐suppressive effect was achieved during the therapeutic process of combining BMS202@HZP NPs and LDRT (Figure 6b). Similarly, it was found that BMS202@HZP NPs‐mediated LDRT could greatly suppress the growth of distant tumors; however, other treatments (X‐ray radiation alone, BMS202@202HP NPs and BMS202@HZP NPs without X‐ray radiation) had only a slight influence on the distant tumors’ growth (Figure 6c,d and Figure S14, Supporting Information). We hypothesized that the enhanced abscopal effect induced by BMS202@HZP NPs‐mediated LDRT potentially inhibited the proliferation of distant tumor tissues. As our hypothesis, the flow cytometry analysis of the distant tumors revealed a significant increase in the number of lymphocytes (CD3+ T cells and CD3+CD8+ T cells) after the BMS202@HZP NPs‐mediated LDRT (Figure 6e,f). We also observed that the expression of Gran B and the number of CD8+ T cells was significantly increased in distant tumors via immunohistochemistry (IHC) staining (Figure 6g), indicating that more activated CD8+ T cells infiltrated into distant tumor and attacked the target tumor cells. In addition, after BMS202@HZP NPs‐mediated LDRT, there were much more CD8+ T cells and CD20+ B cells accumulating in spleen (Figure 6g). Since spleen was one of major peripheral lymphoid organs for lymphocyte activation by antigens, these lymphocytes would be activated and mature in spleen and then recirculate between blood until they encounter their specific antigen.^[^ ^26^ ^]^ To further confirm the abscopal effect, the level of IFN‐*γ* and TNF‐*α* in plasma was measured, which was believed to promote the proliferation and activation of CD8+ T cells. As shown in Figure 6h,i, the single treatment, including 2 Gy X‐ray radiation, BMS202@HP NPs and BMS202@HZP NPs, facilitated TNF‐*α* and IFN‐*γ* expression to some extent due to the immune responses induced by LDRT or BMS202‐induced PD‐1/PD‐L1 blockade, both of which could boost immune cells to produce cytokines. Thus, when combining anti‐PD‐L1 and LDRT through HZP NPs, their synergism would further enhance the level of IFN‐*γ* and TNF‐*α*.

On the other hand, to further evaluate this therapeutic potency on inhibiting lung metastasis of 4T1 cells, the lungs after different treatments were harvested and stained with Bouin's solution and hematoxylin and eosin (H&E). Figure 6j,k reveals that there were 37.2 nodules detected at the lung site in 0.9% NaCl group, while the number of metastatic nodules remarkably decreased to 6.8 after BMS202@HZP NPs‐mediated LDRT, which were also less than those of 2 Gy X‐ray radiation (27.6), BMS202@HP NPs (16.4), and BMS202@HZP NPs (12.2) treatments. These results were further validated by the images of H&E‐stained lung tissues, where both the number and area of lung metastatic nodules in the group of BMS202@HZP NPs‐mediated LDRT were obviously reduced in comparison to other groups (Figure 6l), indicating this synergistically therapeutic strategy could also inhibit the lung metastasis of TNBC. To sum up, BMS202@HZP NPs‐mediated LDRT could induce strong antitumor immunological responses to reinforce the abscopal effect against distant tumor growth and tumor metastasis (Figure 6m).

### 2.7 Biosafety

Biosafety of nanomedicine is an important evaluation criterion for its further clinical application. Thus, we not only evaluated the hemocompatibility of blank HZP NPs but also collected plasma and major organs of mice after different treatments for histopathologic, bodyweight, biochemical, blood routine, and metabolomics analysis. When the blank HZP NPs were incubated with erythrocytes, there only appeared the low hemolysis (<3%) at different concentrations (50–500 µg mL^−1^) (Figure S15, Supporting Information), demonstrating that blank HZP NPs possessed good hemocompatibility. The results of mice body weight, H&E staining images, and biochemical analysis (AST, ALT, CRE, BUN) did not show obvious differences between the BMS202@HZP NPs group and 0.9% NaCl group, indicating that BMS202@HZP NPs could not trigger severe tissue damage and dysfunction in major organs (Figures S16–S18, Supporting Information). However, free BMS202 treatment caused more infiltration of inflammatory cells in kidney. In routine blood test, free BMS202 induced the significant enhancement of WBC and lymph compared to normal group, suggesting it might cause an inflammatory reaction in mice body (Figure S19, Supporting Information). In contrast, the similar symptom was not observed in BMS202@HZP NPs group. Meanwhile, through analyzing the metabolites of serum, there existed five metabolites with statistically significant difference (*p* value < 0.05) between free BMS202 group and normal group (Figure S20 in the Supporting Information), such as the decrease of glycerol and the increase of gluconic acid, myo‐inositol, glycine, and L‐lysine. However, there was only one metabolite (succinic acid) in BMS202@HZP NPs from normal group. Taken together, it is believed that HZP NPs with the controllable UCST would be a suitable carrier for delivering BMS202, which could avoid adverse side effects caused by nonspecific drug leakage and thus achieve the preferable biosafety for further medical application.

## 3 Conclusions

In summary, a multifunctional “drug‐like” copolymer with the auto‐changeable UCST and selective depletion of NADPH inside tumor cells was successfully synthesized and exploited to fabricate a safe and effective nanocarrier for sensitizing radiotherapy and controlled release of antagonist (BMS202) of PD‐1/PD‐L1 interactions. Due to the modification of CD44 receptor HA and hypoxia‐dependent factor CA IX inhibitor Z on the surface of nanocarrier, it achieved the enhanced strike on hypoxic TNBC and mediated LDRT to induce ICD of TNBC, converting the immune “cold” tumor into the “hot” tumor. More importantly, BMS202@HZP NPs‐mediated LDRT significantly promoted the PPP in TME and generated more NADPH to facilitate the polarization of TAMs toward M1‐like phenotype and activation of CD8+ T cells, reprogramming the immunometabolism of TME to alleviate its immunosuppression. Meanwhile, BMS202@HZP NPs were prone to scavenge the NADPH inside tumor cells to impair DNA repair, resulting in the upregulated PD‐L1 expression of tumor cells to reinforce the anti‐PD‐L1 therapy. Based on these advantages, BMS202@HZP NPs‐mediated LDRT achieved the significantly improved synergism effect against TNBC. Thus, it is believed that this nanomedicine‐mediated radio‐immunometabolism regulation would be a promising strategy to reinforce anti‐PD‐L1 against TNBC.

## 4 Experimental Section

The Experimental Section is available in the Supporting Information.

## Conflict of Interest

The authors declare no conflict of interest.

## Supporting information

Supporting Information

## Data Availability

The data that support the findings of this study are available from the corresponding author upon reasonable request.
