# Supplementary material for: NADPH Selective Depletion Nanomedicine‐Mediated Radio‐Immunometabolism Regulation for Strengthening Anti‐PDL1 Therapy against TNBC
Source: Adv Sci (Weinh). 2022 Nov 20;10(3):2203788. doi: 10.1002/advs.202203788 (PMC9875612; doi:10.1002/advs.202203788)
Supplement: Supplementary file 1 — Supporting Information [file ADVS-10-2203788-s001.pdf]

ADVANCED SCIENCE
Open Access

Supporting Information

for *Adv. Sci.*, DOI 10.1002/advs.202203788

NADPH Selective Depletion Nanomedicine-Mediated Radio-Immunometabolism Regulation for Strengthening Anti-PDL1 Therapy against TNBC

Ying Wang, Di Gao, Lin Jin, Xuechun Ren, Yanan Ouyang, Ying Zhou, Xinyu He, Liangliang Jia, Zhongmin Tian, Dingcai Wu* and Zhe Yang**

Supporting Information

NADPH Selective Depletion Nanomedicine-Mediated Radio-Immunometabolism Regulation for Strengthening anti-PDL1 Therapy against TNBC

Ying Wang [†], *Di Gao*[†], *Lin Jin*[†], *Xuechun Ren, Yanan Ouyang, Ying Zhou, Xinyu He, Liangliang Jia, Zhongmin Tian* [*], *Dingcai Wu*[*], *Zhe Yang* [*]

[†] These authors contributed equally to this work.

* Corresponding author

Y. Wang, D. Gao, X. Ren, Y. Ouyang, Y. Zhou, X. He, L. Jia, Z. Tian, Z. Yang

The Key Laboratory of Biomedical Information Engineering of Ministry of Education, School of Life Science and Technology, Xi'an Jiaotong University, Xi'an 710049, China

E-mail: zmtian@mail.xjtu.edu.cn (Z. Tian), yangzhe@xjtu.edu.cn (Z. Yang)

D. Wu

PCFM Lab, School of Chemistry, Sun Yat-sen University, Guangzhou 510006, P. R. China

Center of Accurate Diagnosis, Treatment and Transformation of Bone and Joint Diseases, The Eighth Affiliated Hospital, Sun Yat-sen University, Shenzhen 518000, P. R. China

E-mail: wudc@mail.sysu.edu.cn

L. Jin,

International Joint Research Laboratory for Biomedical Nanomaterials of Henan, Zhoukou Normal University, Zhoukou 466001, P. R. China

Materials, cells, and animals

Acrylonitrile (AN), 2,2′-azoisobutyronitrile (AIBN), acetazolamide (Z), tetrabutylammonium hydroxide (TBA-OH), ion exchange resin (Dowex 50wx8-400), Hoechst 33342, coumarin 6, 3-(4,5-dimethyl-2-thiazolyl)-2,5-diphenyl-2H-tetrazolium bromide (MTT) and concanavalin lectin (ConA) were purchased from Sigma-Aldrich (USA). Acrylamide (AAm) and 2-aminoethanethiol hydrochloride (AET·HCl) were purchased from Shanghai Adamas Reagent Co., Ltd (China). *N*-(2-(2-NI-1H-imidazol-1-yl)ethyl)acrylamide (NIEAAm, NI) was purchased from Jilin province Yanshen Technology Co., Ltd (China). Hyaluronic acid (HA, 60 kDa) was obtained from Shengqiang Biotech Co., Ltd (China). Hexadecylamine (HDA) was purchased from TCI Development Co., Ltd (China). BMS202 was purchased from Med Chem Express (China). DiR was purchased from Biotium Inc. (USA). NADP(H) kit was purchased from the Nanjing Jiancheng Bioengineering Institute (China). The enzymatic activities of glucose-6-phosphate dehydrogenase (G6PD) and 6-phosphogluconate dehydrogenase (6PGD) assay kits were purchased from Solarbio (China). Anti-mouse antibodies: PE-anti-PD-L1, APC-anti-CD3, PE-anti-CD8, APC-anti-CD127, PE-anti-CD25. CD80-FITC, CD86-APC and PE-anti-CD206 were obtained from BioLegend (USA).

RAW264.7 cells (mouse macrophage cells) and 4T1 cells (murine breast cancer cells) were cultured in Roswell Park Memorial Institute (RPMI) and Dulbecco's Modified Eagle's Medium (DMEM), respectively, supplemented with 10% fetal bovine serum (FBS) and 1% penicillin-streptomycin at 37 ^{o}C in a humidified environment with 5% CO_2, which were acquired from the Shanghai Cell Bank of the Chinese Academy of Sciences (China). T cells were isolated using the CD4+ T-cell isolation kit (Miltenyi Biotec, Auburn, CA, USA) from mouse spleen.

The protocol for animal experiments was approved by the Animal Experimentation Ethics Committee of School of Life Science and Technology in Xi'an Jiaotong University. Female

Balb/c mice (5–6 weeks) were purchased and maintained in the Center for Experimental Animals at Xi'an Jiaotong University Health Science Center. Before X-ray radiation treatment, each mouse was anesthetized and shielded by custom lead blocks, such that only the irradiated tumor was exposed. Radiation was performed using a digital accelerator (Elekta Precise) operated at 6 MV, and a dose rate of 600 MU min^{-1}.

The tumor volume was measured via a serial caliper and estimated using Equation 1:

$$Volume = 0.5\times length\times(width)^2 \quad (1)$$

The tumor inhibitory efficiency was calculated by Equation 2:

$$Inhibitory\ efficiency\ (\%) = (1-V_{Sample}/V_{0.9\%\ NaCl}) \times 100\% \quad (2)$$

where V_{Sample} and $V_{0.9\%\ NaCl}$ represented the tumor volume of the mice treated with different drug formulations and 0.9% NaCl, respectively.

Synthesis of PAAN copolymer

The synthesis of poly(acrylonitrile-*co*-acrylamide-*co*-*N*-(2-(2-NI-1H-imidazol-1-yl)ethyl)acrylamide) copolymer (PAAN) was similar to other UCST polymers in our previous study[1]. Briefly, NIEAAm (2.16 mmol), AAm (7.03 mmol) and AN (3.79 mmol) as monomers, AET·HCl (0.11 mmol) as a chain transfer agent and AIBN (0.02 mmol) as an initiator were co-dissolved in anhydrous dimethylformamide (DMF) and transferred into a 25 mL Schlenk flask under argon protection. After degassing via freeze-vacuum-thaw cycles for three times, the reaction was maintained at 65 ^{o}C for 24 h. Finally, PAAN copolymer was precipitated into cold methanol and purified via dialyzing against deionized water followed by lyophilization for further study. Similarly, poly(acrylamide-*co*-acrylonitrile) copolymer (PAA) without NIEAAm was also synthesized as control group. The structures of PAA and PAAN copolymer were characterized using ^{1}H NMR spectrum. ^{1}H NMR (400 MHz, DMSO-d_6) δ (ppm): 7.12–7.51 (m, imidazole and -NH_2), 1.64-2.40 (m, -C$\boldsymbol{H}_2$- in -NIEAAm-, -AAm-, and -AN-), 4.39 (-NH-CH_2-C$\boldsymbol{H}_2$-), 3.46 (-NH-C$\boldsymbol{H}_2$-CH_2-) (Figure S2a). The number-

average molecular weight of PAAN copolymer in DMF was measured using GPC (Agilent PL-GPC50), which was 12266 g mol^{-1}. Besides, the content of NIEAAm in PAAN copolymer was calculated by measuring its characteristic absorbance of UV-vis spectroscopy at a wavelength of 330 nm.

Turbidity measurements of PAAN copolymer

To demonstrate the changeable UCST of PAAN copolymer under hypoxia, the PAAN and PAA copolymer were dispersed in aqueous solution containing NADPH (1mM) and NTR (10 μg mL^{-1}) and incubated at 37 ^{o}C under argon protection for 0, 12 and 24 h, and their turbidities were recorded using a UV-vis spectrophotometer (Purkinje T6-1610F, China) at a wavelength of 650 nm and a heating rate of 0.5 ^{o}C min^{-1}. The UCST of copolymer was defined as the temperature where the transmittance reached half of the maximum value during the whole heating process. In addition, the UV absorbance of PAAN solutions before and after incubation in different solutions was also monitored using a UV-vis spectrophotometer.

Electron affinity measurements of PAAN copolymer

Cyclic voltammetry measurement was employed to evaluate electron affinity of NIEAAm or PAAN copolymer with an equal concentration of nitroimidazole ([NI] = 0.002 M). Meanwhile, n-Bu_4NPF_6 (0.2 M) was selected as the supporting electrolyte. Besides, the Ag/AgCl and platinum electrodes were used as the reference and working electrodes, respectively. The scanning rate was 100 mV s^{-1}.

Synthesis of amphiphilic HA-HDA-Z

The HA-HDA-Z was also synthesized according to our previous protocol.[2] Firstly, HA was converted to its tetrabutylammonium salt (HA-TBA) to improve the solubility of HA in

dimethyl sulfoxide (DMSO). Then, HA-TBA, HDA (0.2 eq. molars of the carboxyl end group of HA), EDC (1.2 eq. molar of HDA) and NHS (1.2 eq. molar of HDA) were co-dissolved in 30 mL of DMSO and then stirred at 35 ^{o}C for 24 h. Subsequently, a solution containing Z-COOH (0.3 eq. molars of hydroxyl end group of HA), EDC (1.2 eq. molar of Z-COOH) and DMAP (1.2 eq. molar of Z-COOH) pre-dissolved in 5 mL of DMSO was added into above solution and stirred at 35 ^{o}C for another 24 h. The resultant mixture was dialyzed against deionized water, and then NaCl (10 mg NaCl per milliliter of solution) was added to the dialysate for ion exchange. Finally, the above solution was precipitated into cold acetone and purified via dialyzing against deionized water for the further use. The HA-HDA was also prepared in similar methods except that there was no grafting of Z-COOH. The ^{1}H NMR spectrum of HA-HDA-Z was as follows: ^{1}H NMR (400 MHz, D_2O) δ (ppm): 1.21 (–CH_2–(C$\boldsymbol{H}_2$)14–CH_3 in HDA), 1.95 (-CO-C$\boldsymbol{H}_3$ in HA), 2.84 (-C$\boldsymbol{H}_2$-C$\boldsymbol{H}_2$- in Z-COOH) (Figure S2b).

Preparation and Characterization of HZP NPs

The blank HZP NPs with dual targeting ligands (HA and Z) were fabricated using a modified emulsification technique. Briefly, PAAN copolymer dissolved in DMSO (250 μL, 20 mg mL^{-1}) was injected into 1 mL of HA-HDA-Z aqueous solution (1 mg mL^{-1}) under probe sonication at 14 W for 1 min on ice (Sonics & Materials, VCX500). Then, the resultant emulsion was transferred into a dialysis bag (MWCO 3500 Da) and dialyzed against deionized water overnight. The blank HP NPs with single targeting ligand (HA) was prepared using a similar procedure except that HA-HDA-Z was replaced with HA-HDA. Meanwhile, to evaluate their drug-loading properties, the targeting capacities and in vivo biodistribution of HZP NPs, the drugs or the fluorescent dyes-loaded NPs were also prepared using the same protocol for blank NPs fabrication, except that the drugs (0.1 mg BMS202) or the fluorescent dyes (0.025 mg coumarin 6 (C6) or 0.15 mg DiR) were co-added to the DMSO solution containing PAAN or PAA copolymer during the HZP or HP NPs preparation, respectively.

Afterward, the size and zeta potential of blank HZP NPs as well as their stability in simulated physiological condition were evaluated using a dynamic light scattering (DLS) analyzer (ZETA-SIZER, NanoZS90, Malvern, Ltd., UK). Meanwhile, the morphology of HZP NPs was observed using a transmission electron microscope (FEI Talos F200C, US).

Measurement of HZP NPs' targeting capacity

4T1 cells at a density of 1.5×10^5 cells well^{-1} were seeded in 24-well plates overnight, and then cultured in normoxic (21% O_2) or hypoxic (1% O_2) condition for another 24 h. Then, the C6-loaded NPs ([C6] = 0.5 μg mL^{-1}) were incubated with 4T1 cells for 4 h. In competitive inhibition experiments, the cells were pre-incubated with HA (10 mg mL^{-1}) or Z (0.2 mg mL^{-1}) for 1 h at 37 ^{o}C followed by C6-loaded HZP or HP NPs incubation. Moreover, to further demonstrate the high selectivity of HZP NPs to 4T1 cells under hypoxia, we incubated coumarin 6 (C6)-loaded HZP NPs with 4T1 cells, RAW264.7 macrophages and T cells under normoxia and hypoxia for 4 h, respectively. Subsequently, the fluorescence intensity of cells was quantitatively detected by flow cytometry (Becton Dickinson, San Jose) at an excitation wavelength of 488 nm. Meanwhile, the HZP NPs targeted to 4T1 cells were also directly visualized using Confocal Laser Scanning Microscopy (CLSM, Olympus FV3000).

Entrapment efficiency of BMS202 encapsulated in HZP NPs

The content of BMS202 encapsulated in HZP NPs was determined by high performance liquid chromatography (HPLC) using a C18 column (25 mm × 4.6 mm, 5 μm) on Agilent 1100 system. The column was eluted using a mixture of different gradient ratios of acetonitrile and water at 30 ^{o}C with a flow rate of 1.0 mL min^{-1}. The content of BMS202 was monitored with a UV detector according to their absorbance at 230 nm. The entrapment efficiency of BMS202 loading in NPs was calculated using Equation 3:

Entrapment efficiency (%) = drug amount in NPs / amount of drug used ×100% (3)

In vitro drug release profiles

To investigate in vitro drug release profiles, BMS202@HZP NPs were added into dialysis bags (MWCO:14 kDa) and then immersed into PBS (pH 7.4, 0.01 M) at 37 °C. In addition, NADPH (1 mM) and NTR (10 μg mL^{-1}) was added in external solution under argon protection to mimic hypoxic condition. At predetermined time intervals, the external solution was removed and the dialysate was replenished by fresh solution with the same volume. The amount of released BMS202 was determined using HPLC as described above.

Blocking PD-L1 receptor efficacy

Flow cytometry measurement was utilized to quantitatively evaluate the affinity of BMS202@HZP NPs with the PD-L1 protein. Briefly, 4T1 cells at a density of 3×10^5 cells $well^{-1}$ were seeded in 6-well plates for 24 h. Then, the cells were treated with free BMS202 and BMS202@HZP NPs ([NI] = 238 μM, [BMS202] = 2.57 μg mL^{-1}), which were incubated for another 24 h under hypoxic conditions. Subsequently, the cells were washed with PBS, then incubated with PE-anti-PD-L1 followed by flow cytometry detection.

Selective NADPH depletion

To evaluate the nanomedicine's capacity of selective scavenging NADPH inside tumor cells, 4T1 cells and RAW264.7 cells at a density of 6×10^5 cells $well^{-1}$ were seeded in 6-well plates for 12 h. Then, the cells were incubated with blank HZP NPs and BMS202@HZP NPs ([NI] = 238 μM, [BMS202] = 2.57 μg mL^{-1}) for 6 h under hypoxic or normoxic conditions, followed by 0 or 2 Gy of X-ray radiation. After another 12 h of incubation, the cells were harvested to measure the intracellular NADPH/$NADP^+$ ratio.

MTT assay

4T1 cells at a density of 5×10^3 cells $well^{-1}$ were seeded in 96-well plates for 24 h. Then, the cells were incubated with free BMS202, blank HZP NPs and BMS202@HZP NPs at varying

concentrations of NI (0, 24, 48, 119, 238 and 357 μM) and BMS202 (0.26, 0.51, 1.28, 2.57 and 3.85 μg mL^{-1}) for 6 h under hypoxic and normoxic conditions. Subsequently, the cells were irradiated with 2 Gy of X-ray radiation and followed by another 24 h of incubation. Subsequently, the cell viability was assessed using MTT assay. The synergistic effect was further evaluated by calculating the combination index (CI) with Equation 4:

$$CI = D_1/D_{1s} + D_2/D_{2s} \quad (4)$$

where D_1 and D_2 are the IC_{50} values of BMS202 and NI in BMS202@HZP NPs group, and D_{1s} and D_{2s} are the IC_{50} values of free BMS202 and NI in blank HZP NPs group, respectively. In addition, immunogenic cell death (ICD) induced by different drug formulations with or without X-ray radiation were also evaluated. Briefly, 6×10^5 of 4T1 cells were treated with free BMS202, blank HZP NPs and BMS202@HZP NPs ([NI] = 238 μM, [BMS202] = 2.57 μg mL^{-1}) for 6 h under hypoxic and normoxic conditions, followed by 0 or 2 Gy of X-ray radiation. After another 24 h of incubation, 4T1 cells were collected to evaluate the CRT expression using western blot. The intensities of bands were quantified by ImageJ.

Colony formation assay

For colony formation assay, 4T1 cells at a density of 800 cells $well^{-1}$ were seeded in 6-well plates for 24 h. Then, the cells were treated with free BMS202, blank HZP NPs and BMS202@HZP NPs ([NI] = 238 μM, [BMS202] = 2.57 μg mL^{-1}) for 6 h under hypoxic and normoxic conditions, which were then exposed to X-ray radiation at a dose of 2 Gy. Additionally, the experiment was performed for 7 days and the media were changed every other day. At the end of experiment, the cell clones were immobilized with methanol, stained with crystal violet and photographed. Finally, the stained cells were decolorized with 30% acetic acid, and the absorbance of decolonization solution at 540 nm wavelength was measured using a microplate reader (Tecan M200) to quantitatively estimate the cell clone rate.

In vitro immune regulation

To evaluate the in vitro immune regulation of BMS202@HZP NPs, a double-layered cell model was built using a transwell (the pore size: 0.4 μm). Briefly, a mixed cell composed of PBMC and 4T1 cells at a ratio of 50:1 were co-seeded in the upper chamber of the transwell under ConA (100 μg mL^{-1}) stimulation, and the lower chamber were also seeded with 4T1 cells at density of 2×10^4 cells $well^{-1}$. The co-cultured PBMC/4T1 cells were then treated with BMS202@HZP NPs ([NI] = 238 μM, [BMS202] = 2.57 μg mL^{-1}) under hypoxic and normoxic conditions for 6 h. Afterwards, the upper cells were irradiated with X-rays at a dose of 2 Gy. After another 24 h of incubation, the alive 4T1 cells in lower chamber were stained with calcein-AM and photographed using an inverted microscope (Olympus IX53). Meanwhile, the supernatant in the lower chamber was also collected for TNF-α and IFN-γ determination using ELISA assay.

In vivo biodistribution studies

For HZP NPs' biodistribution evaluation, subcutaneous 4T1 tumor were cultured on the back of female mice. When the tumor size reached to 400∼ 500 mm^3, the 4T1 tumors were treated with or without 2Gy X-ray radiation followed by intravenous injection of free DiR and DiR-loaded HZP and HP NPs at the dye dose of 0.35 mg kg^{-1} mouse body weight. At pre-determined time intervals (2, 4, 6, 8, 12, and 24 h), the fluorescent images of these mice were captured using in vivo imaging system (MAG Biosystems Lumazone, USA). At the end of experiment, the mice were sacrificed and the major tissues (heart, liver, spleen, lung, kidney and tumor) were harvested for *ex vivo* imaging.

Additionally, the tumor-bearing mice pre-treated with or without 2 Gy of X-ray radiation were injected (i.v.) with 0.9% NaCl, BMS202@HP NPs and BMS202@HZP NPs at a dose of 1 mg BMS202 per kg body weight of mouse, respectively. At 24 h post-injection, the mice

were immediately injected with Evans blue solution. After another 24 h, all mice were subjected to PBS perfusion, and tumor tissues were harvested and photographed. Finally, all tumor tissues were dried and weighed, and the Evans blue was then extracted from tumor using DMF, which was quantified by determining the absorbance at 620 nm.

In vivo therapeutic efficiency assessment

When the subcutaneous tumor on the right flank of Balb/c mice grew to ~150 mm^3, the mice were randomly divided into five groups: (1) 0.9% NaCl; (2) 0.9% NaCl +2 Gy LDRT; (3) BMS202@HP NPs; (4) BMS202@HZP NPs; (5) BMS202@HZP NPs +2 Gy LDRT. Different drug formulations were injected (i.v.) into Balb/c mice on day 1, 4, and 7. At 24 h post-injection, the tumors were exposed to X-ray at a dose of 2 Gy. The tumor size, body weight, and survival rate were monitored throughout the whole experiment period. Besides, on day 10, a part of mice in each group was sacrificed, and the tumors and plasma were collected to evaluate the immune responses.

For RNA sequencing analysis, total RNA from tumor tissues was extracted. Libraries were subsequently constructed using Standard Illumina Novaseq. Transcriptome sequencing and analysis were performed by Haorui Genomics Co, Ltd (Xi'an, China). Besides, the tumors were collected to evaluate the mechanisms of immune regulation. Briefly, the metabolites of tumor tissues were analyzed by gas chromatography-mass spectrometry (GC-MS). Briefly, the extraction buffer (methanol/water/chloroform = 2.5:1:1) was added to tumor tissues at a ratio of 1:10 (w/v). Subsequently, the extracts were subjected to oximation and derivatization. Finally, the metabolites in tumor tissues were measured by Agilent 7890A gas chromatograph and 5975C mass spectrometer (Agilent Technologies, Wilmington, DE). In addition, the enzymatic activities of G6PD and 6PGD and the level of NADPH and $NADP^+$ in tumor were also measured according to the manufacturer's protocols of assay kits.

For flow cytometry, the single-cell suspension from the harvested tumor tissue was utilized to detect the amount of $CD3^+CD8^+$ T cells and mature DCs in tumors, and the ratios of $CD8^+$ T cells *vs.* T_{regs} and M1 *vs.* M2. Briefly, the eviscerated tumor tissues were cut into small pieces and then digested in type I collagenase (1 mg mL^{-1}) at 37 °C for 4 h. The resultant cells were filtered through a 200-mesh sieve filter and washed with PBS three times. The cells were blocked using 3% BSA and further stained with the following fluorochrome-conjugated antibodies: APC-anti-CD3, PE-anti-CD8, APC-anti-CD127, PE-anti-CD25, FITC-anti-CD80, APC-anti-CD86 and PE-anti-CD206 according to the manufacturer's protocol, respectively. Those cells were rinsed with PBS for three times and analyzed using flow cytometry analysis. Meanwhile, the collected tumor tissues were used for IHC (PD-L1, CD8 and granzyme B) and IF analysis (F4/80, CD86 and CD206). At the end of experiment (on 21 days post-injection), all the mice were sacrificed and the tumors were collected to perform H&E staining and IHC (CD8 and granzyme B) analysis.

Abscopal effect and anti-lung metastasis efficacy

The 4T1 bilateral tumor model was established to evaluate abscopal effect and anti-lung metastasis efficacy of BMS202@HZP NPs-mediated LDRT. Briefly, 1×10^6 4T1 cells were injected (*s.c.*) in the right flank of Balb/c mice as primary tumor. 7 days later, 1×10^5 4T1 cells were injected in the left flank of Balb/c mice as distant tumor. When the primary tumors reached ~150 mm^3, all mice were randomly divided into five groups with the same treatment as mentioned above, and the primary tumor was treated with 2 Gy of X-ray radiation. The volumes of primary and distant tumors were monitored throughout the whole experiment period. At the end of experiment, the primary and distant tumor, spleen and plasma were collected for H&E, IHC analysis (CD8, granzyme B and CD20) and ELISA assay (IFN-γ and TNF-α), respectively. Besides, the lungs were also harvested and stained by Bouin's solution

(picric acid/formaldehyde/acetic acid = 15:5:1) and H&E to further evaluate the anti-lung metastasis efficacy.

Biosafety studies

To study the biosafety of NPs, the hemolysis tendency of blank HZP and HP NPs was evaluated. Briefly, the erythrocytes at the density of 1×10^{7} cells mL^{-1} were incubated with blank HP NPs and HZP NPs at concentrations ranging from 50 µg mL^{-1} to 500 µg mL^{-1} at 37 °C for 12 h. PBS (pH 7.4, 0.01 M) and Triton X-100 (1%, v/v) solution were also selected as negative and positive control, respectively. The absorbance of hemoglobin at the wavelength of 410 nm in the supernatant was measured using the microplate reader (Tecan M200). Hemolytic activity (%) was calculated using Equation 5:

$$\text{Hemolysis (\%)} = (A_{Sample} - A_{PBS})/(A_{Triton} - A_{PBS}) \times 100\% \quad (5)$$

where A_{Sample}, A_{PBS}, A_{Triton} represent the absorbance intensity of hemoglobin in blank HZP and HP NPs, PBS, and Triton X-100, respectively.

Furthermore, the biosafety of free BMS202 and BMS202@HZP NPs were also estimated in vivo. In brief, the major organs (heart, liver, spleen, lung and kidney) from mice treated with free BMS202 and BMS202@HZP NPs were harvested and stained with H&E after 21 days of treatment. Additionally, the blood was also collected for the routine blood examination and the plasma was obtained for biochemistry analysis, including aspartate transaminase (AST), creatinine (CRE), alanine aminotransferase (ALT), and urea nitrogen (BUN). Meanwhile, the metabolomics of serum was also analyzed by GC-MS using the same treatment as mentioned above, except that the extraction buffer was a mixture of isopropanol, acetonitrile and water in a ratio of 3:3:2 (v/v/v).

Statistical analysis

All of the data are reported as the mean ± standard deviations (SD) from at least three repeated experiments. Statistical analysis were performed using a two-sided Student's t-test unless otherwise indicated (Graphpad Software). *$p < 0.05$ and **$p < 0.01$ were considered to be statistically significant and extremely significant, respectively.

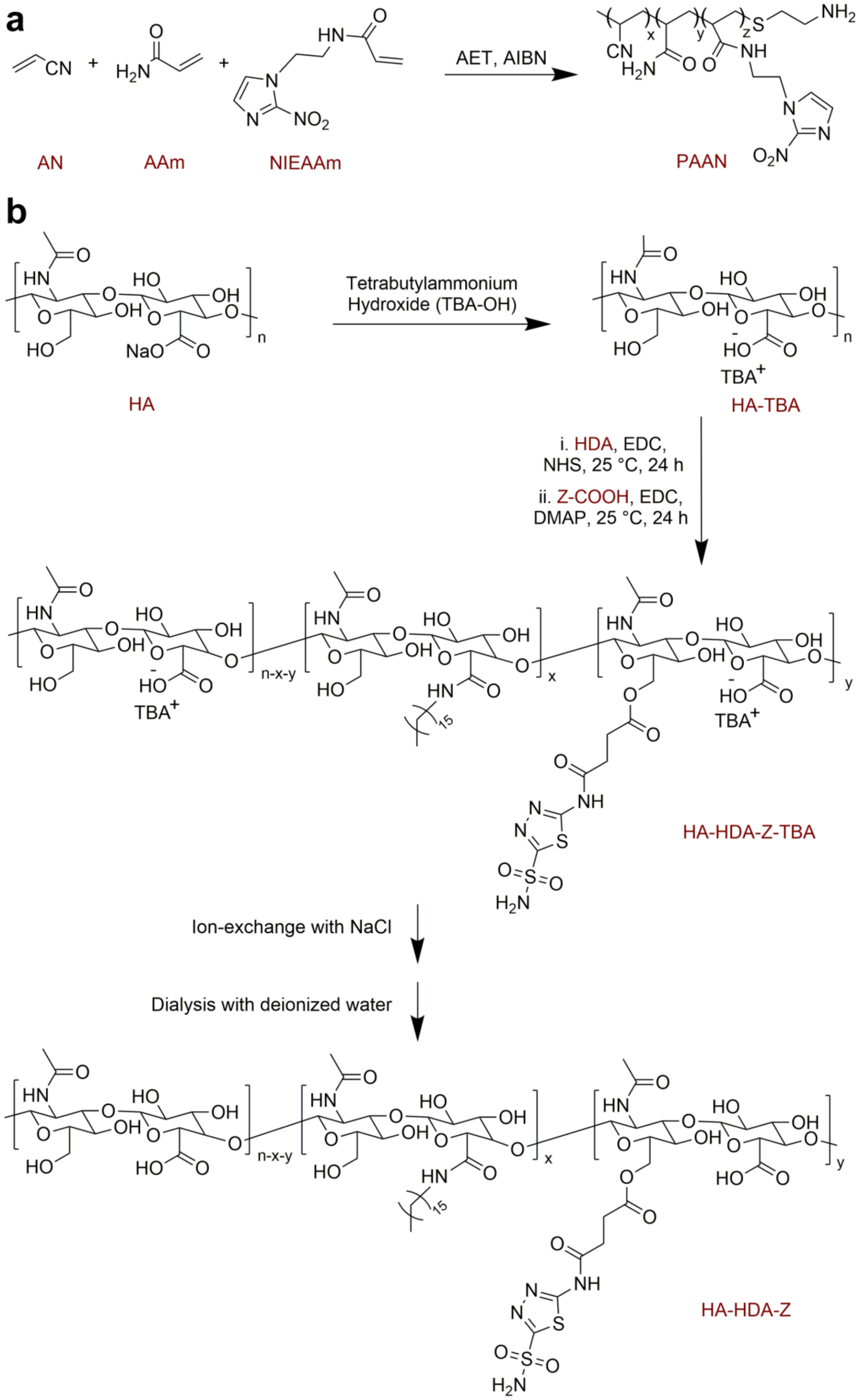

Figure S1. Synthesis of P(AN -*co*-AAm-*co*-NIEAAm) copolymer (PAAN) (a) and amphiphilic HA-HDA-Z (b).

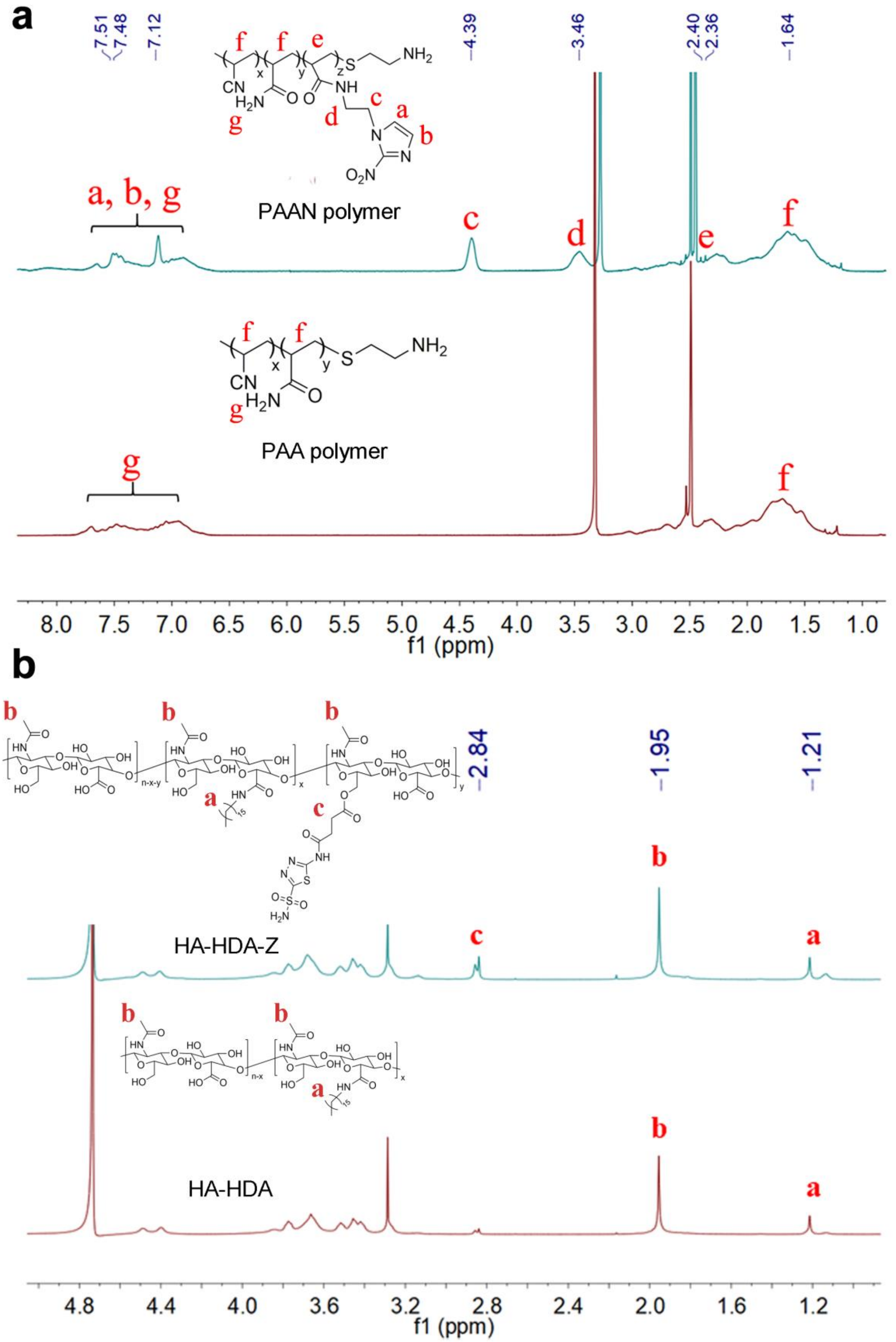

Figure S2. ^{1}H-NMR spectrums of PAAN and PAA copolymer in DMSO-d_6 (a) and HA-HDA-Z and HA-HDA in D_2O (b).

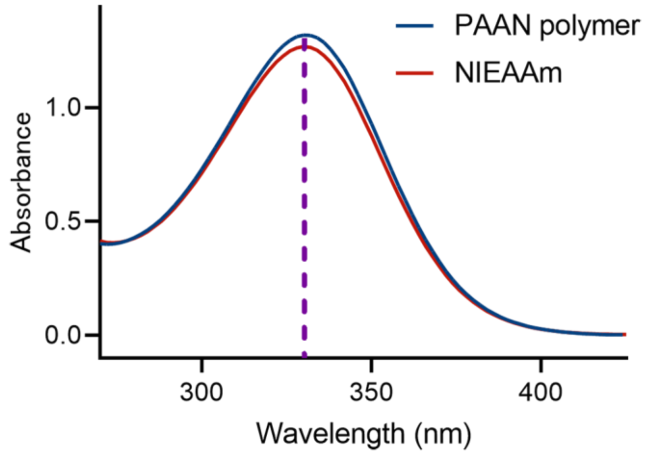

Figure S3. UV-VIS spectra of NIEAAm and PAAN polymer in DMSO.

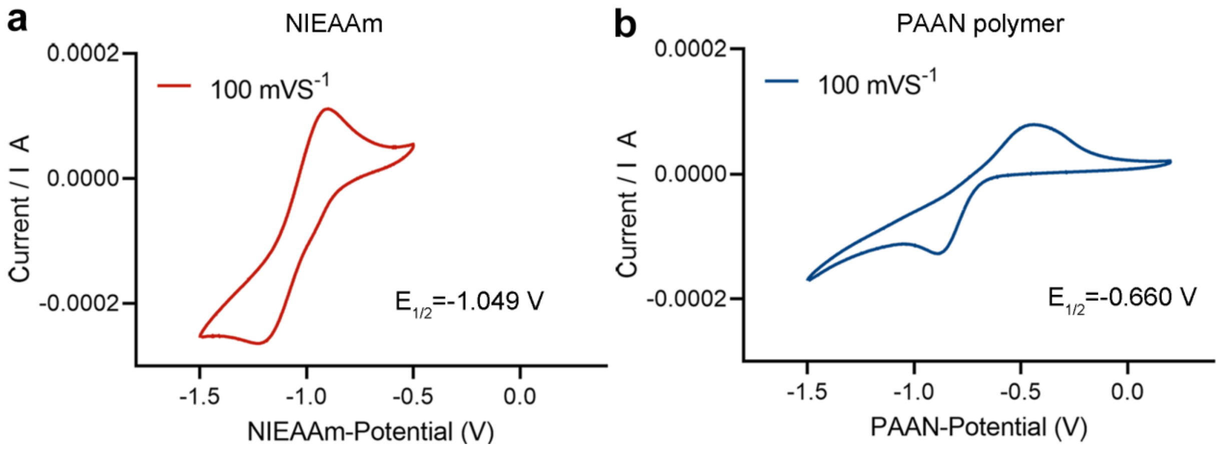

Figure S4. Cyclic voltammogram of NIEAAm (a) and PAAN polymer (b) at the scan rate of 100 mVS^{-1}.

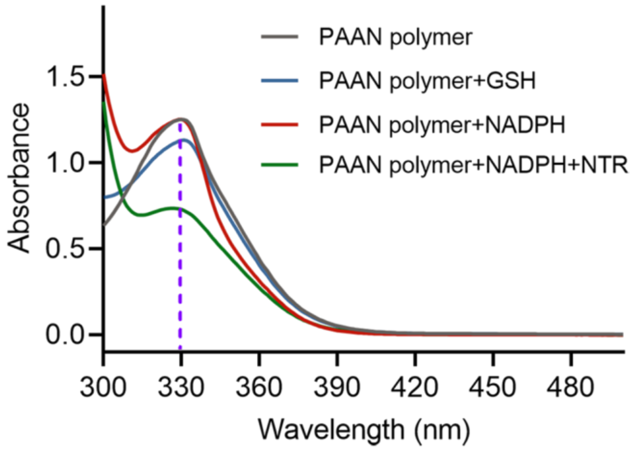

Figure S5. UV-VIS spectrums of the PAAN polymer before and after incubation under hypoxic conditions containing GSH (2 mM), NADPH (1 mM) or NADPH (1 mM) with NTR (10 μg mL^{-1}) for 24 h.

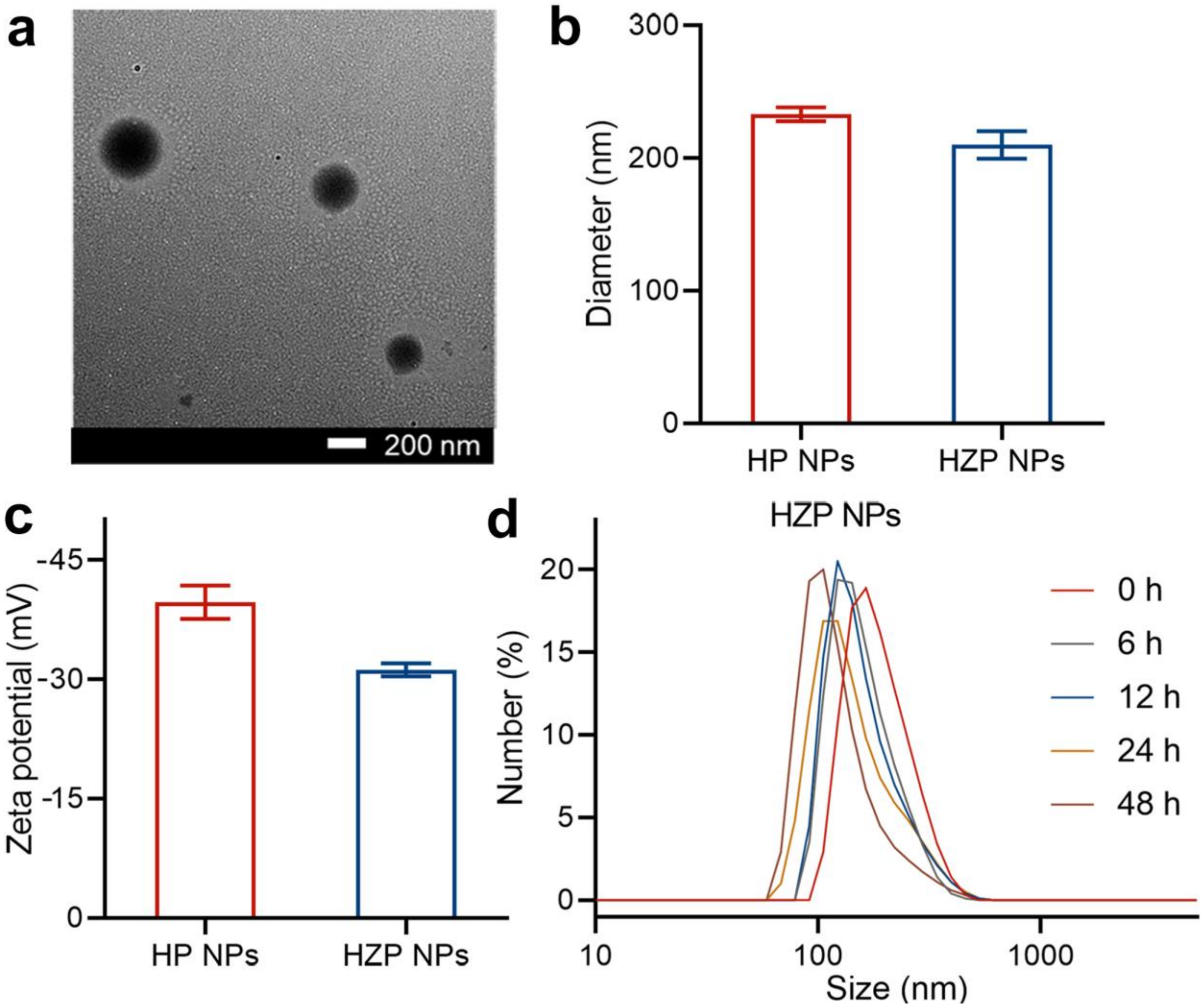

Figure S6. a) TEM image of HZP NPs. b,c) Characterization of different NPs, including hydrodynamic diameters (b) and ζ-potential (c). d) Hydrodynamic diameter distribution of HZP NPs after incubation in DMEM with 10% FBS at different intervals at 37 ^{o}C. (mean ± SD, n = 3)

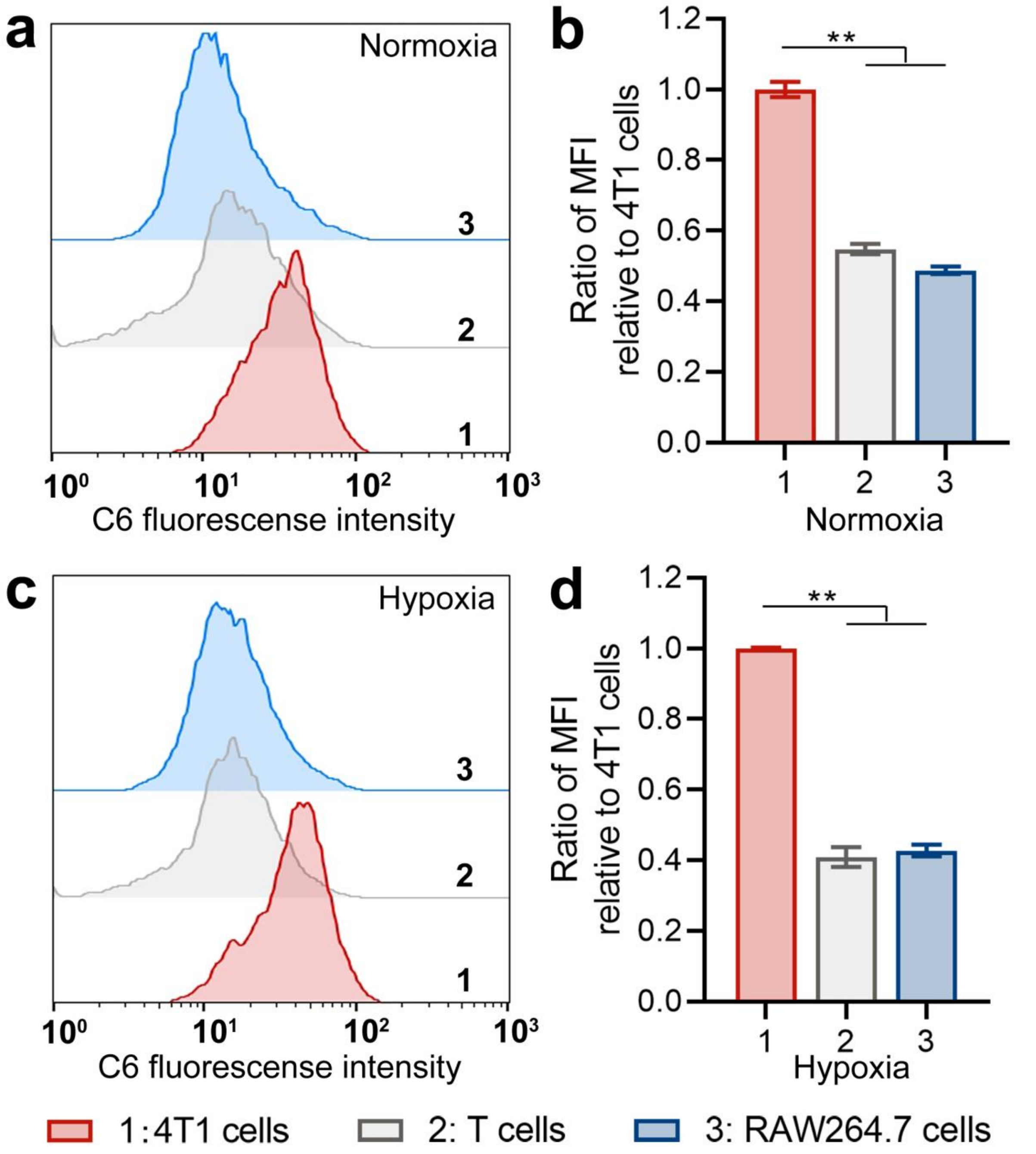

Figure S7. Targeting capacity of HZP NPs. a-d) Fluorescence histograms (a,c) and mean fluorescence intensities (b,d) of different cells incubated with HZP NPs/C6 under normoxia and hypoxia. (mean ± SD, n = 3, $**p < 0.01$)

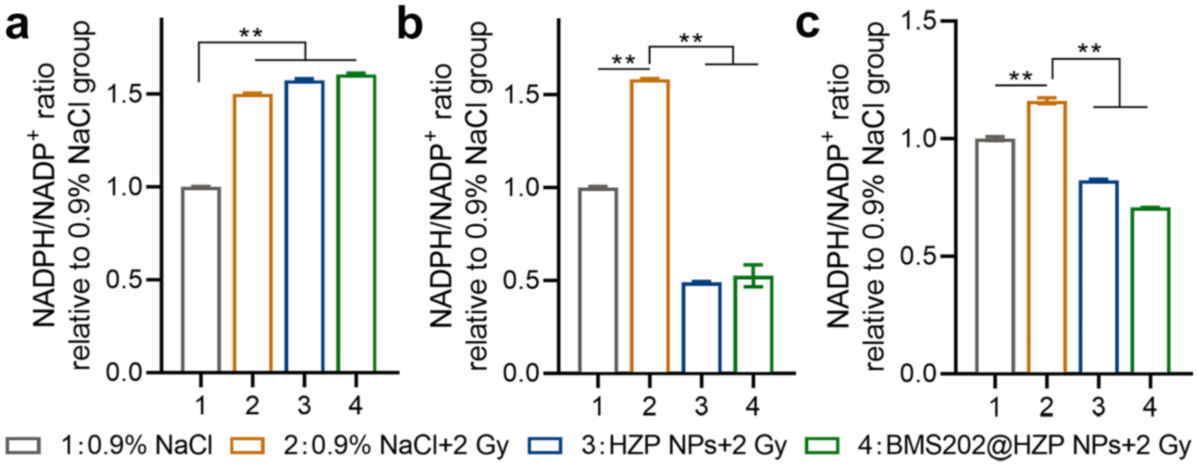

Figure S8. Relative NADPH/NADP$^+$ ratio in RAW264.7 cells under normoxia (a) and 4T1 cells under normoxia (b) and hypoxia (c) after the treatment of 0.9% NaCl, 0.9% NaCl+2 Gy of X-ray, blank HZP NPs+2 Gy of X-ray and BMS202@HZP NPs+2 Gy of X-ray. (mean ± SD, n = 3, $^{**}p < 0.01$)

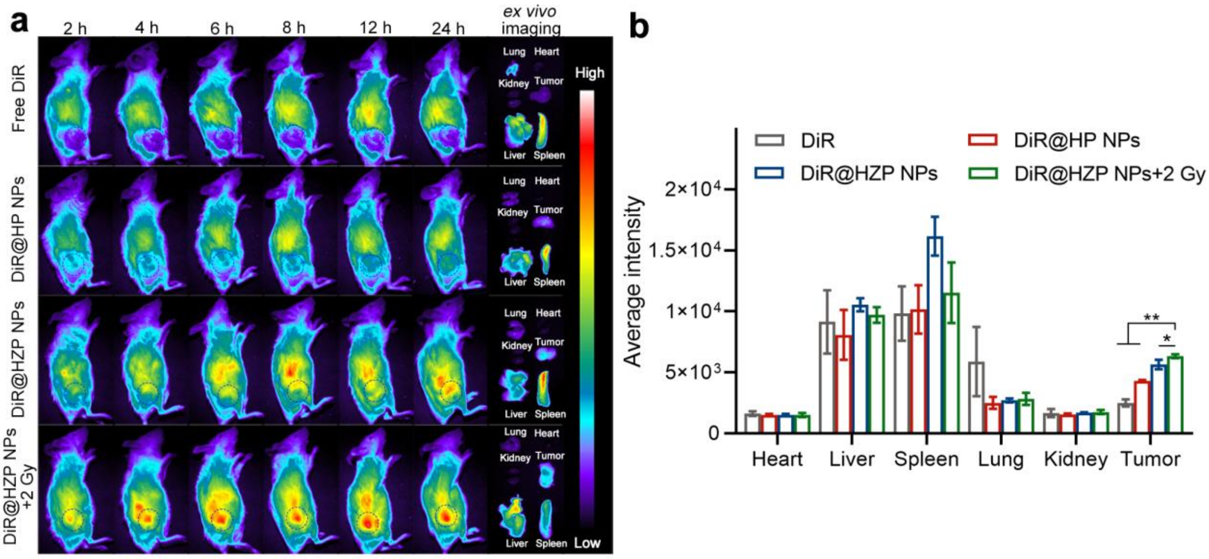

Figure S9. a) DiR fluorescence imaging of Balb/c mice bearing subcutaneous 4T1 tumors at 2 h, 4 h, 6 h, 8 h, 12h and 24 h post-injection of different drug formulations with or without 2 Gy X-ray radiation and *ex vivo* NIR fluorescence imaging of DiR fluorescence intensity in the harvested organs and tumors at 24 h post-administration. **b**) Quantitative analysis of DiR fluorescence intensity in the harvested organs and tumors at 24 h post-administration. (mean ± SD, n = 3, $^{*}p < 0.05$, $^{**}p < 0.01$)

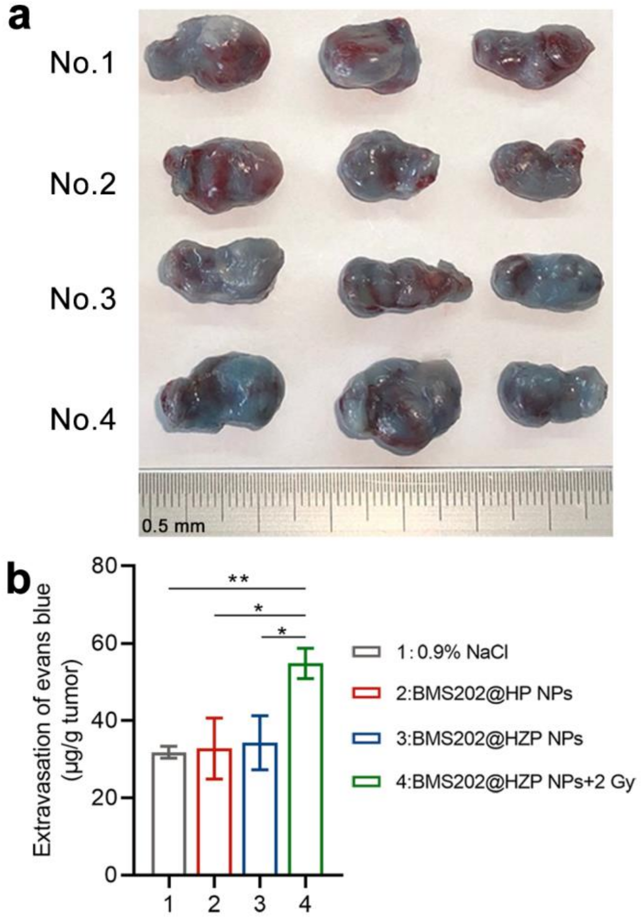

Figure S10. Image (a) and quantitative analysis (b) of leaking of Evans blue dye in the 4T1 tumor after the treatments of 0.9% NaCl, BMS202@HP NPs and BMS202@HZP NPs with or without 2 Gy of X-ray radiation. (mean ± SD, n = 3, *$p < 0.05$, **$p < 0.01$)

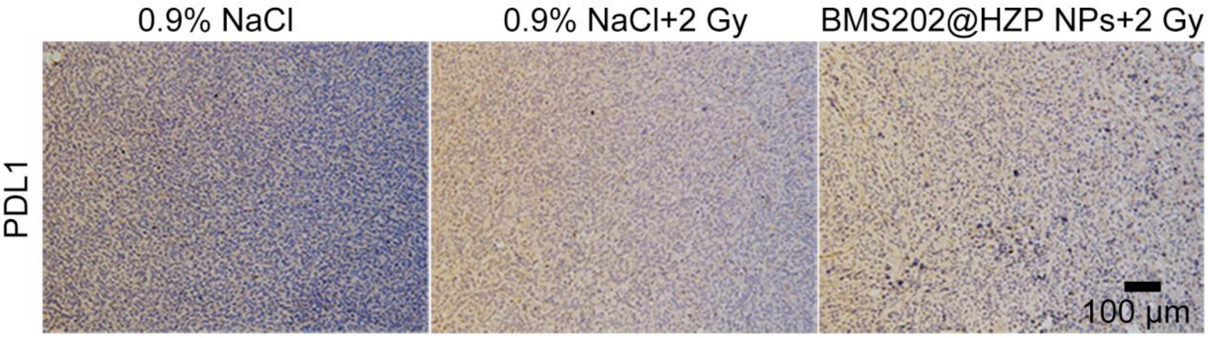

Figure S11. IHC staining for PD-L1 of tumor tissue in Balb/c mice bearing 4T1 tumors following the treatments of 0.9% NaCl with or without 2 Gy of X-ray radiation and BMS202@HZP NPs with 2 Gy of X-ray radiation.

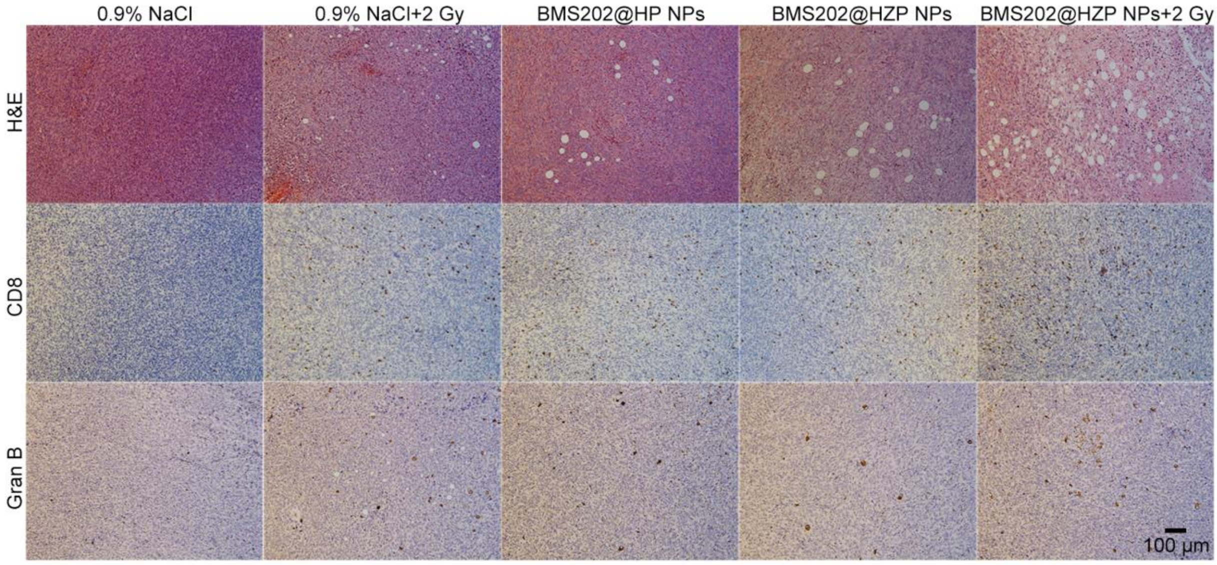

Figure S12. H&E images and IHC staining of CD8+ T cell and Gran B of tumor tissue in Balb/c mice bearing 4T1 tumors following the treatments of BMS202@HP NPs, 0.9% NaCl and BMS202@HZP NPs with or without 2 Gy of X-ray radiation.

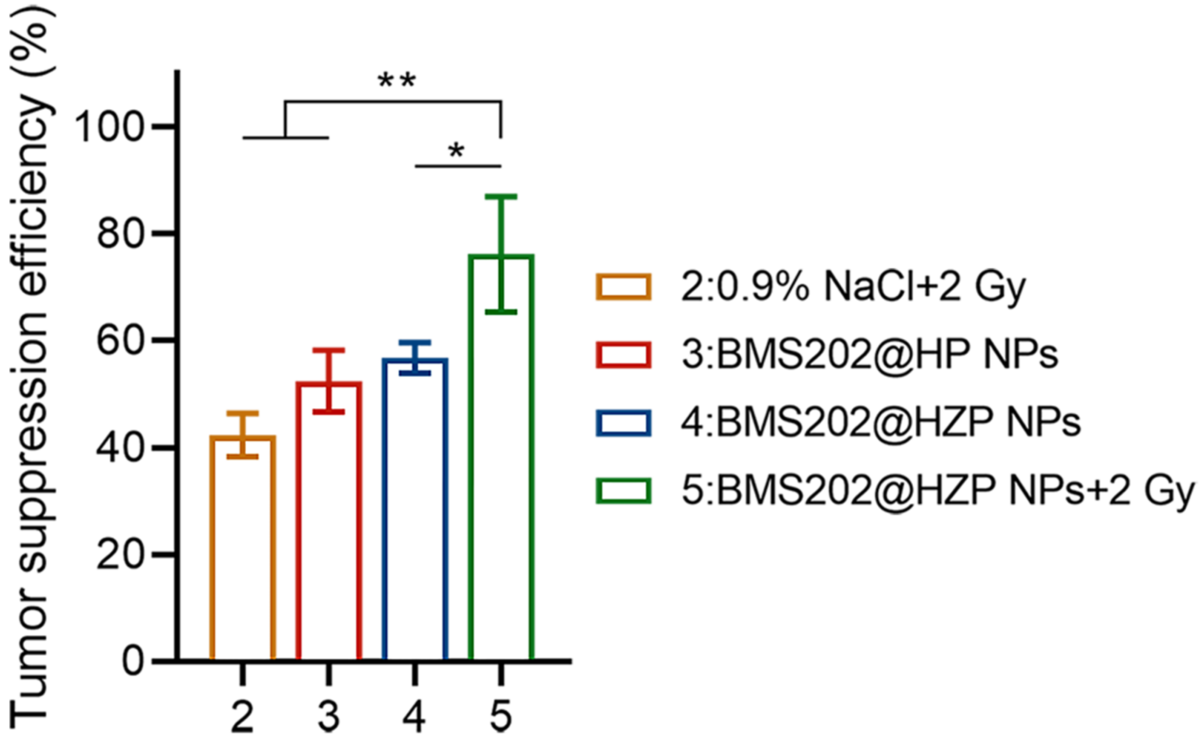

Figure S13. Tumor inhibitory efficiency in Balb/c mice bearing 4T1 tumors following the treatments of 0.9% NaCl with 2 Gy of X-ray radiation, BMS202@HP NPs and

BMS202@HZP NPs with or without 2 Gy of X-ray radiation. (means ± SD, n = 5, *$p < 0.05$, **$p < 0.01$)

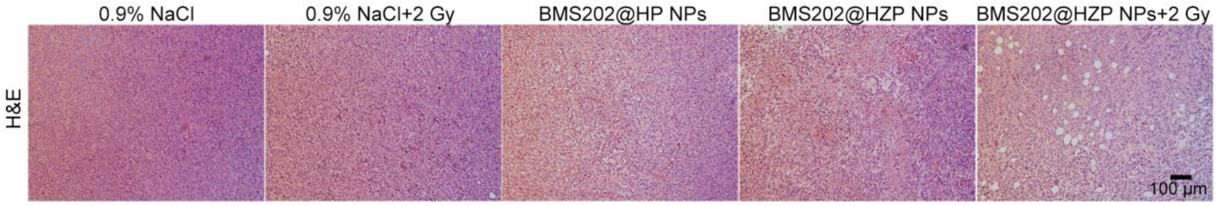

Figure S14. H&E images of distant tumors in Balb/c mice bearing 4T1 bilateral tumors following the treatments of BMS202@HP NPs, 0.9% NaCl and BMS202@HZP NPs with or without 2 Gy of X-ray radiation.

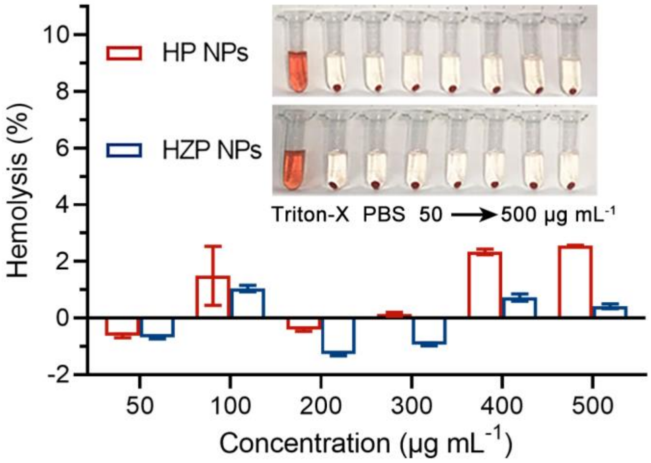

Figure S15. Hemolytic activity of blank HP NPs and HZP NPs with varying concentrations (50, 100, 200, 300, 400 and 500 μg mL^{-1}) after incubation with erythrocytes for 12 h. (mean ± SD, n = 3)

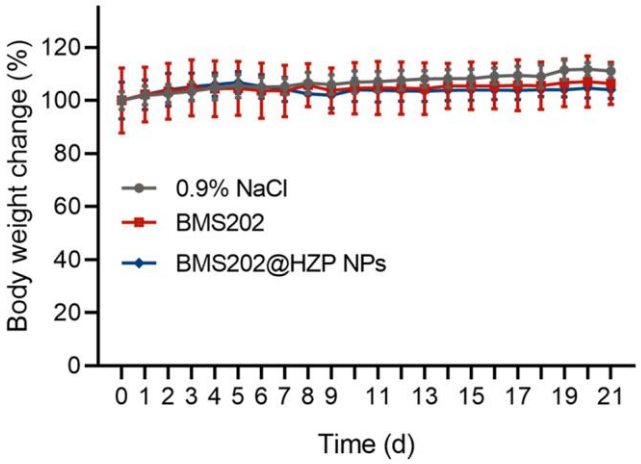

Figure S16. The changes in body weight during the experimental period (0~21 day). (mean ± SD, n = 4)

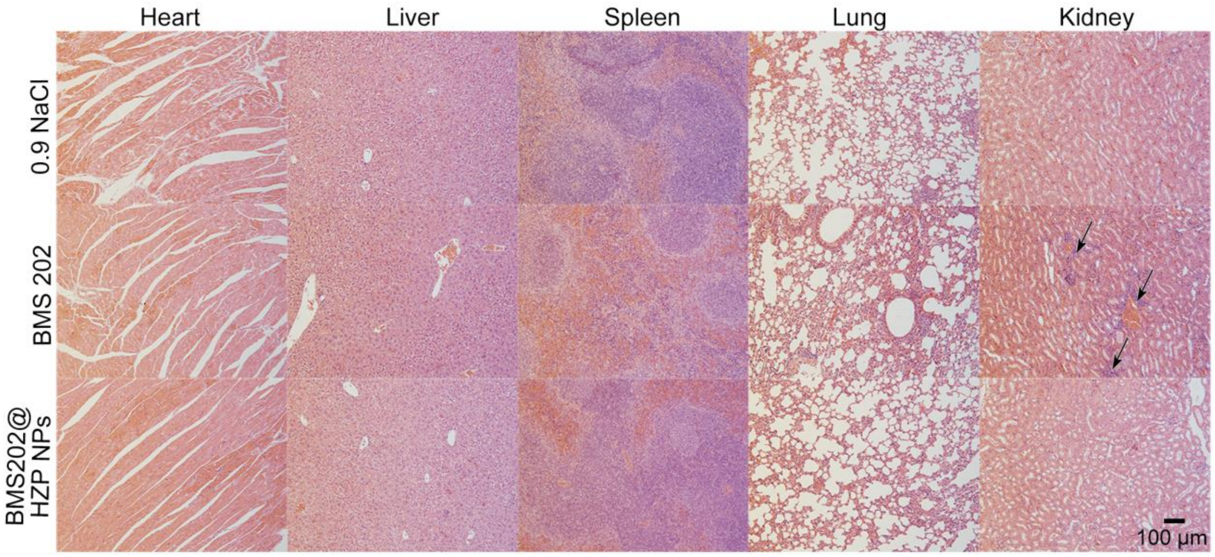

Figure S17. H&E images of the different organs in Balb/c mice after systematic injection of 0.9% NaCl, free BMS202 and BMS202@HZP NPs.

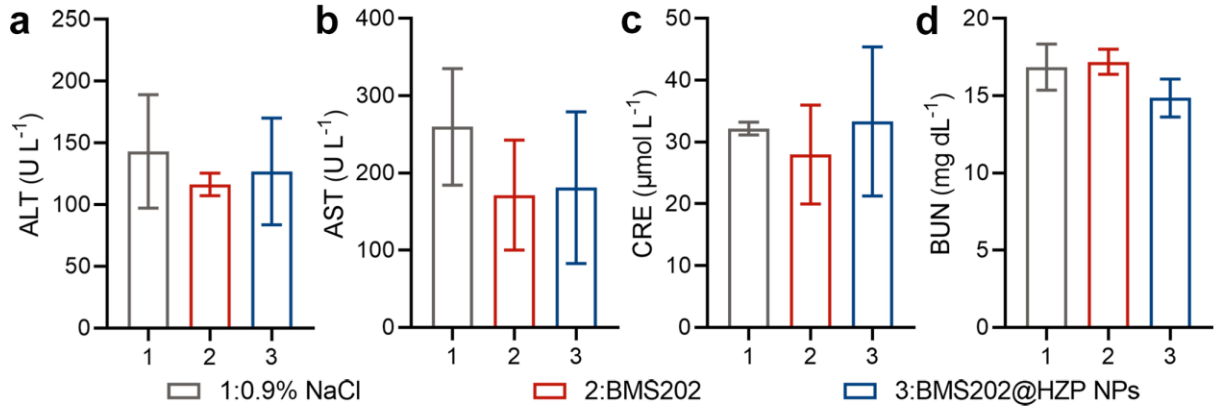

Figure S18. The blood level of ALT (a), AST (b), CRE (c) and BUN (d) in plasma from Balb/c mice after systematic injection of 0.9% NaCl, free BMS202 and BMS202@HZP NPs. (mean ± SD, n = 4)

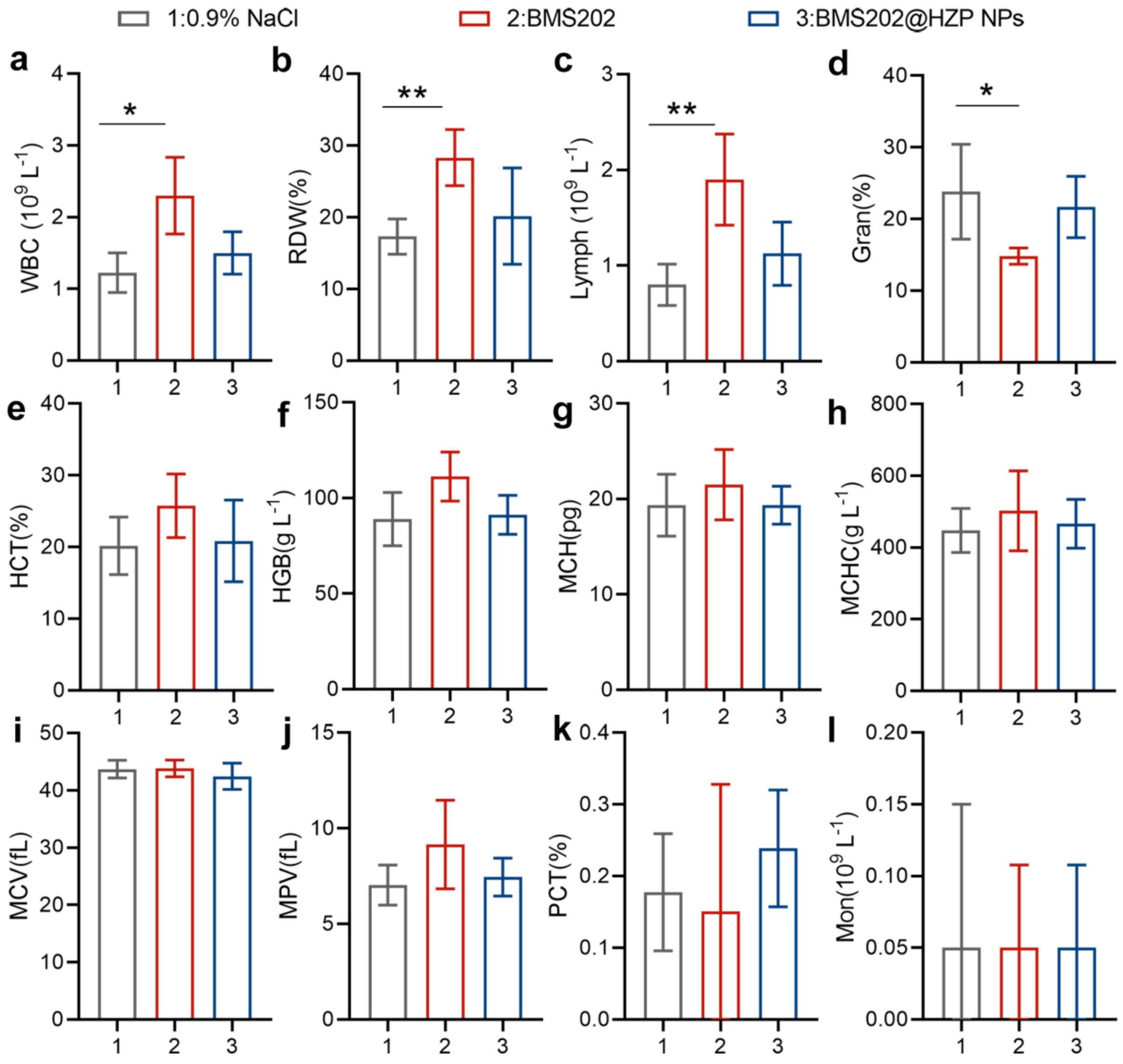

Figure S19. The routine blood examination of Balb/c mice after systematic injection of 0.9% NaCl, free BMS202 and BMS202@HZP NPs. (mean ± SD, n = 4, *$p < 0.05$, **$p < 0.01$)

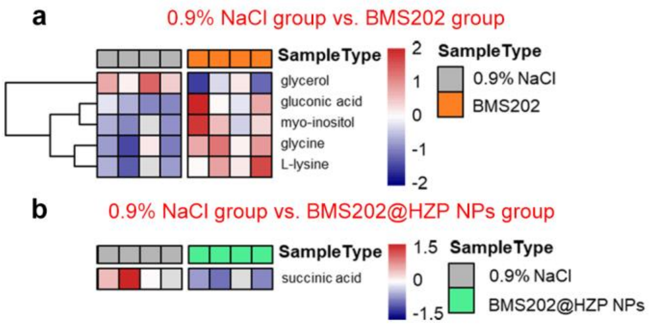

Figure S20. Heatmap clustering analysis of the differential metabolites in serum between free BMS202 (a) or BMS202@HZP NPs+2 Gy group (b) and 0.9% NaCl group (p < 0.05) (n = 4).

Table S1. IC_{50} values of free BMS202, blank HZP NPs and BMS202@HZP NPs with or without 2 Gy of X-ray radiation.

Condition	Free BMS202	HZP NPs	BMS202@HZP NPs
	[BMS202][a)]	[NI group][b)]	[BMS202][a] / [NI group][b]
Normoxia	1.08	-[c)]	0.37 / 34.73
Normoxia+2 Gy	0.76	442.09	0.24 / 22.69
Hypoxia	2.24	-[c)]	1.38 / 128.6
Hypoxia+2 Gy	2.67	39869.96	1.12 / 104.46

[a)] The concentration unit for BMS202 is μg mL^{-1}; [b)] The concentration unit for NI group is μM; [c)] It could not be determined up to the concentration of 357 μM of NI group.

Table S2. The metabolites in tumor for the groups of BMS202@HZP NPs + LDRT and 0.9% NaCl (n ≥ 5).

Number	Compound Name	FC (BMS202@HZP NPs+2 Gy *vs.* 0.9% NaCl)
1	phosphoric acid	1.4518693
2	glycine	0.730677704
3	L-(+) lactic acid	1.119134043
4	stearic acid	0.308210499
5	glycerol 1-phosphate	3.051435593
6	palmitic acid	0.346747765
7	fumaric acid	1.058095546
8	myo-inositol	1.504263478
9	citric acid	0.543212101
10	hypotaurine	0.839673947
11	O-phosphocolamine	0.783740553
12	dihydroxyacetone phosphate	0.639975045
13	D-glucose	0.291785737
14	glyceric acid	0.364894625
15	D-glucose-6-phosphate	0.143566815
16	D-malic acid	0.907244777
17	Beta- alanine	0.838211102
18	xylitol	1.082393745
19	gluconic acid	0.449255784

20	DL-isoleucine	0.628002339
21	L-alanine	0.910350004
22	L-serine	0.4156435
23	pipecolic acid	1.210926075
24	aspartic acid	0.677714173
25	L-valine	0.620699357
26	glycolic acid	0.927760116
27	pyrophosphate	2.122425952
28	oleic acid	0.169266689
29	2-hydroxypyridine	1.363559794
30	L-lysine	0.446617856
31	pantothenic acid	0.904782999
32	L-proline	0.710928841
33	putrescine	2.084428445
34	D-threitol	0.470005973
35	trans-4-hydroxy-L-proline	1.150896004
36	thymine	0.445409244
37	L-threonine	0.576731185
38	L-tyrosine	0.437775277
39	Creatinine	2.493759397
40	ribulose-5-phosphate	3.716785524
41	urea	0.950734315
42	L-cysteine	0.653917597
43	Sucrose	0.774569976
44	xanthine	0.337607494
45	cellobiose	2.396071395
46	cholesterol	0.092949524
47	inosine	6.0492332
48	L-leucine	0.988747205
49	orotic acid	0.507226075
50	adenosine	3.301304407
51	D-ribose-5-phosphate	5.722186082
52	guanosine	3.740947702

Table S3. The differential metabolites in tumor for the groups of BMS202@HZP NPs + 2 Gy and 0.9% NaCl (both VIP > 1 and FDR-adjusted *p* value < 0.05, n ≥ 5).

Compound Name	log2(FC)	VIP	Raw *p*	FDR adjust *p*
2-hydroxypyridine	0.447378	1.477444	0.000709	0.006376
adenosine	1.723036	1.386723	0.002596	0.016874
Creatinine	1.318322	1.519306	0.000263	0.00601
D-glucose-6-phosphate	-2.80021	1.501209	0.000347	0.00601
D-ribose-5-phosphate	2.516566	1.467805	0.000681	0.006376
glyceric acid	-1.45445	1.206936	0.013817	0.044906
glycerol 1-phosphate	1.609488	1.458292	0.000771	0.006376
inosine	2.596752	1.507648	0.000315	0.00601
L-serine	-1.26658	1.451483	0.000858	0.006376
L-threonine	-0.79403	1.33953	0.005252	0.024825
L-tyrosine	-1.19174	1.360095	0.003318	0.01917
ribulose-5-phosphate	1.894055	1.292	0.005785	0.025068

Reference

[1] a) Y. Wang, D. Gao, Y. Liu, X. Guo, S. Chen, L. Zeng, J. Ma, X. Zhang, Z. Tian, Z. Yang, *Bioact. Mater.* **2021**, *6*, 1513; b) Z. Yang, R. Cheng, C. Zhao, N. Sun, H. Luo, Y. Chen, Z. Liu, X. Li, J. Liu, Z. Tian, *Theranostics* **2018**, *8*, 4097.

[2] D. Gao, T. Chen, S. Chen, X. Ren, Y. Han, Y. Li, Y. Wang, X. Guo, H. Wang, X. Chen, M. Guo, Y. S. Zhang, G. Hong, X. Zhang, Z. Tian, Z. Yang, *Nanomicro. Lett.* **2021**, *13*, 99.
